# Optimized ACE2 decoys neutralize antibody-resistant SARS-CoV-2 variants through functional receptor mimicry and treat infection in vivo

**DOI:** 10.1126/sciadv.abq6527

**Published:** 2022-12-07

**Authors:** James A. Torchia, Alexander H. Tavares, Laura S. Carstensen, Da-Yuan Chen, Jessie Huang, Tianshu Xiao, Sonia Mukherjee, Patrick M. Reeves, Hua Tu, Ann E. Sluder, Bing Chen, Darrell N. Kotton, Richard A. Bowen, Mohsan Saeed, Mark C. Poznansky, Gordon J. Freeman

**Affiliations:** ^1^ Department of Medical Oncology, Dana-Farber Cancer Institute and Harvard Medical School, Boston, MA 02215, USA.; ^2^ National Emerging Infectious Diseases Laboratories (NEIDL), Boston University, Boston, MA 02118, USA.; ^3^ Department of Biochemistry, Boston University School of Medicine, Boston, MA 02118, USA.; ^4^ Center for Regenerative Medicine of Boston University, Boston Medical Center, and The Pulmonary Center and Department of Medicine, Boston University School of Medicine, Boston, MA 02118, USA.; ^5^ Division of Pediatrics, Harvard Medical School, Boston, MA 02115, USA.; ^6^ Vaccine and Immunotherapy Center, Massachusetts General Hospital and Harvard Medical School, Boston, MA 02129, USA.; ^7^ LakePharma Inc., San Carlos, CA 94070, USA.; ^8^ Department of Biomedical Sciences, Colorado State University, Fort Collins, CO 80523, USA.

## Abstract

As severe acute respiratory syndrome coronavirus-2 (SARS-CoV-2) evolves to escape natural antibodies, it also loses sensitivity to therapeutic antibody drugs. By contrast, evolution selects for binding to ACE2, the cell-surface receptor required for SARS-CoV-2 infection. Consistent with this, we find that an ACE2 decoy neutralizes antibody-resistant variants, including Omicron, with no loss in potency. To identify design features necessary for in vivo activity, we compare several enzymatically inactive, Fc effector–silenced ACE2-Fc decoys. Inclusion of the ACE2 collectrin-like domain not only improves affinity for the S protein but also unexpectedly extends serum half-life and is necessary to reduce disease severity and viral titer in Syrian hamsters. Fc effector function is not required. The activity of ACE2 decoy receptors is due, in part, to their ability to trigger an irreversible structural change in the viral S protein. Our studies provide a new understanding of how ACE2 decoys function and support their development as therapeutics to treat ACE2-dependent coronaviruses.

## Teaser

ACE2 decoys refold and inactivate the viral S-protein, and ACE2 homodimerization is important for their in vivo activity.

## INTRODUCTION

The coronavirus disease 2019 (COVID-19) pandemic has resulted in hundreds of millions of cases and millions of deaths worldwide. Vaccines and monoclonal antibodies have been successfully deployed for COVID-19 prophylaxis and treatment, but the emergence of viral variants that are resistant to vaccines and therapeutics remains an ongoing concern (*1*). Multiple resistant variants have emerged, including the B.1.351 (Beta), P.1 (Gamma), B.1.617.2 (Delta), and B.1.1.529.1 to B.1.1.529.5 (Omicron BA.1 to BA.5) variants (*2*–*8*). As severe acute respiratory syndrome coronavirus-2 (SARS-CoV-2) becomes endemic, evolution under the selective pressure of vaccination and natural immunity leads to additional variants (*1*, *3*, *4*, *7*, *8*). Since much of the selective pressure imparted by humoral immunity is focused on the S protein and in particular on the receptor binding domain (RBD), continued evolution of SARS-CoV-2 reduces the efficacy of therapeutic monoclonal antibodies, which also bind the RBD. Consistent with this, the Beta and Delta SARS-CoV-2 variants demonstrated resistance to some antibodies developed for COVID-19 treatment and prophylaxis. The subsequent Omicron BA.1 variant was resistant to almost all of these antibodies, including casirivimab, imdevimab, etesivimab, bamlanivimab, tixagevimab, cilgavimab, ADG10, adintrevimab (ADG20), and ADG30 (*2*, *3*). The more recent Omicron BA.2, BA.3, BA.4, and BA.5 variants are also resistant to these antibodies, as well as to sotrovimab (*8*). The U.S. Food and Drug Administration (FDA) has therefore placed restrictions on the use of each of these antibodies. There is also concern that SARS-CoV-2 may eventually develop resistance to Paxlovid, the oral antiviral that was most effective in trials and is most widely used for the treatment of COVID-19 (*9*). Recent reports have identified naturally occurring viral polymorphisms at Paxlovid’s binding site that can reduce its effectiveness, and it remains to be seen whether such polymorphisms will be selected for in the wake of increasingly widespread use (*10*–*13*). Thus, there is a need for a broadly active therapeutic capable of treating resistant viral variants as they continue to arise as well as future novel coronaviruses that might enter the human population.

SARS-CoV-2 uses angiotensin-converting enzyme 2 (ACE2) on the surface of cells as a receptor for viral entry. ACE2 is a type I transmembrane protein with an extracellular domain composed of a membrane-distal peptidase domain (PD) that cleaves and inactivates angiotensin-II and also contains the binding site for the RBD of coronavirus S proteins and a membrane-proximal collectrin-like domain (CLD) that mediates ACE2 homodimerization (*14*, *15*). ACE2 decoy receptors, which are recombinant soluble forms of the ACE2 protein, have been proposed as therapeutics to treat SARS-CoV-2 infection (*16*–*26*). Similar to antibodies, these decoy receptors engage the RBD of the viral S protein and are thought to outcompete cell-surface ACE2 for viral binding, thus inhibiting viral entry and infection (*17*, *19*, *24*, *25*). Unlike antibodies, the viral binding domain of the decoy is, in theory, identical to the cell-surface receptor, making it difficult for resistant variants to emerge because mutations that reduce affinity of the virus for the decoy receptor drug would also reduce viral affinity to cell-surface ACE2 and reduce infectivity. This broad activity profile should provide lasting antiviral activity as SARS-CoV-2 variants continue to arise. Moreover, several human coronaviruses and numerous coronaviruses with other mammalian hosts that have the potential to become zoonoses are ACE2 dependent (*27*–*29*). The frequency of cross-species viral transmission is expected to increase as changes in climate and land use continue globally (*30*). ACE2 decoys may therefore be useful against novel coronaviruses that emerge in the human population in the future.

Studies of ACE2 decoy receptors have primarily focused on their capacity to neutralize virus in vitro (*16*–*20*, *22*, *23*, *25*). Those that have investigated in vivo activity have mostly used ACE2 PD mutants selected to improve binding affinity to a particular SARS-CoV-2 viral S protein (*21*, *24*, *31*, *32*). Use of an altered ACE2 PD introduces structural differences between the viral binding domain of the drug and the natural receptor that may lead to loss of drug binding by viral variants, undermining the principle of soluble decoy receptors. In addition, nonnative, structurally altered proteins are often immunogenic, raising concern for the development of anti-ACE2 antibodies that might both impair drug activity and cross-react with endogenous cell-surface ACE2. Last, the breadth of activity against other ACE2-dependent coronaviruses, including novel coronaviruses yet to arise in the human population, may be negatively affected by the use of mutated ACE2 PDs that have been selected for their ability to bind better to a single ACE2-dependent virus.

Design features that might improve the in vivo activity of an ACE2-Fc decoy without mutation of ACE2 surface residues are less well studied. For instance, the ACE2 CLD has been reported to improve the affinity of ACE2-Fc decoys for the S protein in vitro (*18*, *19*, *23*). However, it is unclear why this is the case since the CLD does not directly bind virus. It is also unknown whether inclusion of the CLD in an ACE2-Fc decoy has additional consequences for its function and whether it is important for in vivo antiviral activity.

In addition, the mechanism by which ACE2 decoys neutralize virus has yet to be thoroughly investigated. It has been presumed that ACE2 decoys function as competitive inhibitors that must remain bound to the S protein to block its binding to cell-surface ACE2 (*17*, *19*, *24*, *25*). However, ACE2 binding to the S protein can trigger irreversible refolding of the S protein in an event that is important for fusion of the viral membrane with the host cell membrane (*33*–*35*). When this occurs, the S protein loses its ability to bind ACE2. The implications of this for viral neutralization by ACE2 decoys remain unclear. It is not known whether ACE2 decoys trigger the premature refolding of the SARS-CoV-2 S protein and whether this affects the potency of ACE2 decoy–mediated inhibition of the S protein–ACE2 interaction.

In this study, we test the hypothesis that neutralization of virus by an ACE2 decoy is not affected by naturally selected mutations in the S protein that impart resistance to antibodies. We compare ACE2-Fc decoy designs to identify elements that improve S-protein binding and viral neutralization but do not involve mutation of ACE2 surface residues. Our experimental results and structural analysis offer an explanation for why inclusion of the ACE2 CLD enhances the apparent affinity for S protein of ACE2-Fc decoys. We also find that the CLD also unexpectedly improves the pharmacokinetics of ACE2-Fc decoys and is required for their in vivo antiviral activity. We find that Fc effector function is not required for the in vivo activity of ACE2-Fc decoys. Last, we offer insight into the mechanism of ACE2-Fc decoys by discovering that their binding can trigger irreversible refolding of the viral S protein from the prefusion state, which can bind cell-surface ACE2, to the postfusion state, which cannot. This S-protein inactivation enhances the potency of ACE2-Fc decoy–mediated inhibition of the S protein–ACE2 interaction.

## RESULTS

### ACE2 decoys potently neutralize antibody-resistant SARS-CoV-2 variants, including Omicron

**Fig. 1.**
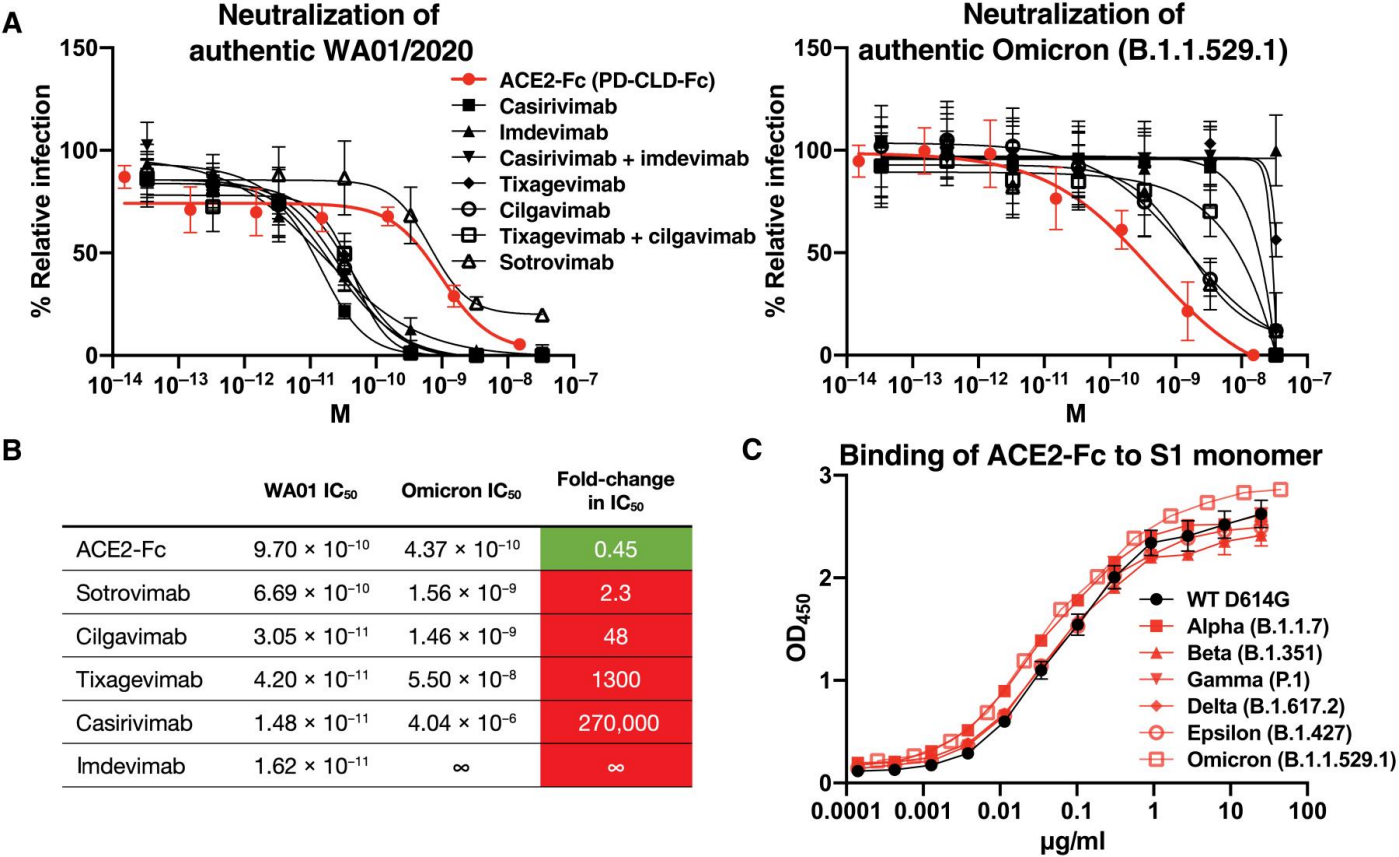
An ACE2-Fc decoy neutralizes the Omicron variant more potently than antibodies and loses no binding affinity to variants of concern. (**A**) Neutralization by an ACE2-Fc (PD-CLD-Fc) or antibodies of infection of ACE2-transduced A549 cells by the original SARS-CoV-2 WA01 isolate or the Omicron BA.1 (B.1.1.529.1) variant. Cells were infected with a multiplicity of infection of 0.5. (**B**) Comparison of IC_50_ against WA01 and Omicron for the ACE2-Fc and each of the antibodies tested. (**C**) The binding of soluble S1 monomer from the D614G parent, Alpha (B.1.17), Beta (B.1.351), Gamma (P.1), Delta (B.1.617.2), Epsilon (B.1.427), and Omicron BA.1 (B.1.1.529.1) to immobilized PD-CLD-Fc was measured by enzyme-linked immunosorbent assay (ELISA).

To test the hypothesis that ACE2 decoys are not susceptible to naturally selected mutations in the S protein that impart resistance to antibodies, we assessed the ability of an ACE2-Fc containing the complete ACE2 extracellular domain (PD-CLD-Fc) to neutralize the SARS-CoV-2 Omicron (B.1.1.529.1) variant, which is resistant to most antibodies that have been developed for COVID-19 treatment and prophylaxis (*3*). We found that this ACE2-Fc neutralized authentic Omicron virus with a median inhibitory concentration (IC_50_) in the subnanomolar range and was 50% more potent in neutralizing Omicron than in neutralizing the original WA01/2020 isolate (Fig. 1, A and B). In contrast, we found that all tested antibodies neutralized Omicron less potently than WA01/2020 (Fig. 1, A and B). The ACE2-Fc neutralized Omicron more potently than each of these antibodies, including sotrovimab, cilgavimab, and tixagevimab, the antibodies with some remaining activity against Omicron BA.1 (Fig. 1, A and B).

We also assessed the ability of PD-CLD-Fc to neutralize the SARS-CoV-2 Beta (B.1.351) variant, which is resistant to many FDA-authorized monoclonal antibodies (*2*, *36*). PD-CLD-Fc neutralized Beta variant pseudovirus and D614G pseudovirus with similar potency (fig. S1A). In addition, we found that PD-CLD-Fc bound to a recombinant S1 monomer of all variants tested with equivalent or higher affinity than to D614G (Fig. 1C and fig. S1B). These results demonstrate that, unlike antibodies, an ACE2 decoy maintains its neutralization potency against SARS-CoV-2 as the virus evolves under the selective pressure of humoral immunity.

### Rational design of ACE2-Fc fusions to identify optimal design parameters

**Fig. 2.**
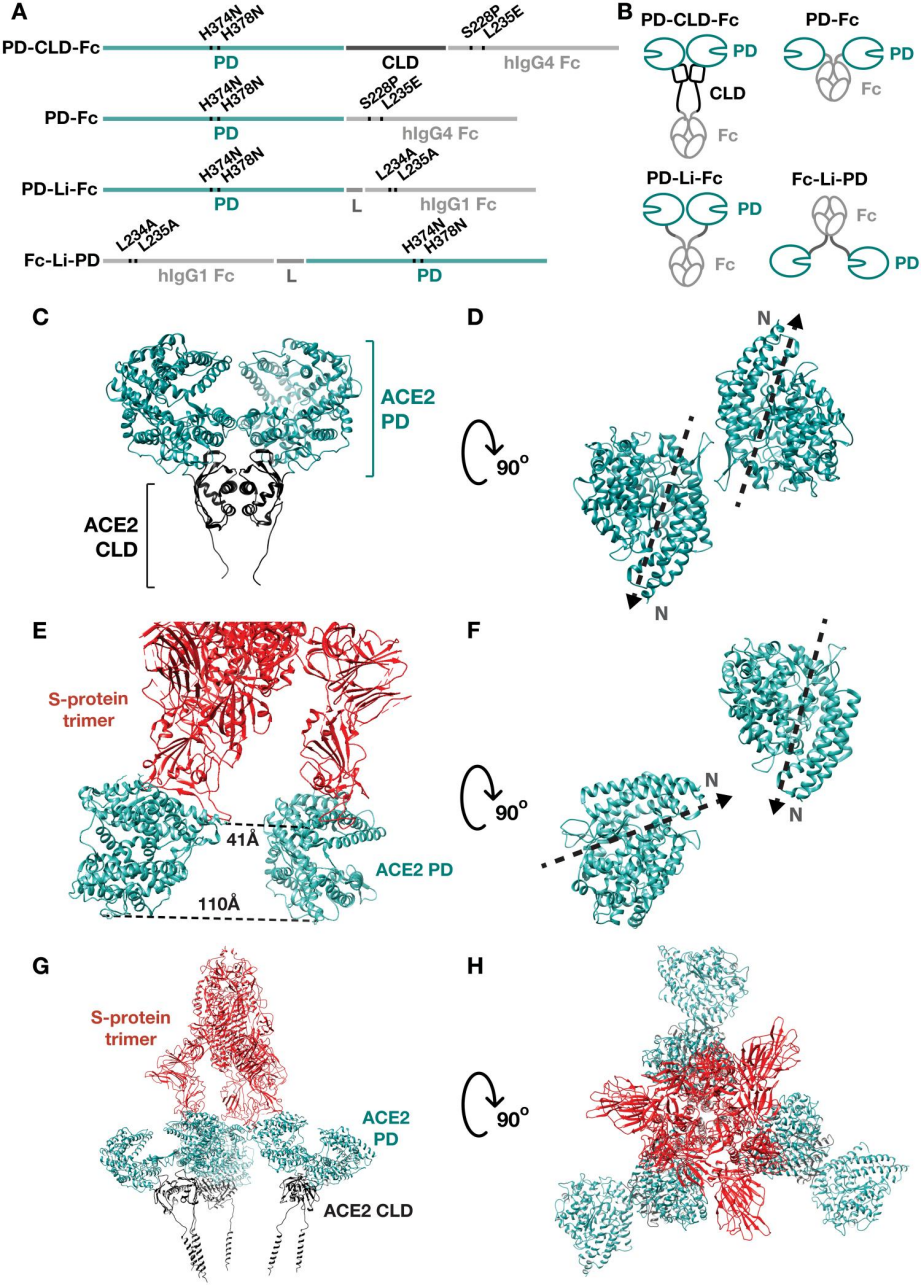
Rational design of ACE2-Fc decoys to investigate structure-function relationships. (**A** and **B**) Four ACE2-Fc fusions were generated to assess the importance of the ACE2 CLD, ACE2/Fc domain orientation, and hinge flexibility on S-protein affinity, viral neutralization, and in vivo antiviral activity. (**C**) The structure of the ACE2 extracellular domain (6M18) with the PD highlighted in cyan and the CLD in black. (**D**) A top-down view of PDs in structure 6M18 shows that the PDs of an ACE2 dimer are in an antiparallel orientation, with their N termini pointing away from one another. (**E**) Structure 7KJ3 of two ACE2 PDs binding simultaneously to two RBDs in a single S-protein trimer shows that their C termini are more than twice the distance from one another compared to their N termini. (**F**) A top-down view of two RBD-bound PDs in 7KJ3 shows that they are oriented with their N termini pointing toward one another. (**G**) A composite of structure 7A98 and 6M18 showing the anticipated orientation of three full-length ACE2 dimers binding a single S-protein trimer. One PD from each ACE2 dimer (6M18) was superimposed on each of the three S-protein–bound PDs in structure 7KJ3 by minimizing RMSD. (**H**) A top-down view of this structure shows that three ACE2 EC dimers bind without steric hindrance.

To identify ACE2-Fc protein design features that optimize binding to S protein and viral neutralization while avoiding the introduction of potentially immunogenic mutations in the ACE2 sequence, we designed four fusion proteins that include different ACE2 subdomains and are linked in different orientations with respect to the Fc domain (Fig. 2, A and B). We termed these PD-CLD-Fc, PD-Fc, PD-Li-Fc, and Fc-Li-PD, where Li indicates a linker. In each, we included a two–amino acid substitution that silences the catalytic activity of ACE2 to mitigate the risk of hypotension from inhibition of the renin-angiotensin-aldosterone system (RAAS), with the goal of allowing high serum concentrations to be well tolerated (fig. S2A). The two mutated histidine residues are responsible for chelating a zinc atom that is necessary for angiotensin II hydrolysis (fig. S2B). We also included point substitutions in the Fc domain to attenuate Fc receptor binding and thereby mitigate the risk of antibody-dependent enhancement (ADE) of viral infection, an immunopathologic process by which Fcγ receptor–expressing immune cells are infected with virus and for which Fcγ receptor binding is necessary (*37*–*39*). All ACE2-Fc exhibited markedly reduced or no binding to FcγRI, FcγRIIa, and FcγRIIIa (fig. S3).

In designing each ACE2-Fc, we considered how its structure might affect its affinity for and thus ability to neutralize the natural S-protein trimer, which contains three RBDs. In particular, we considered whether the design of dimeric ACE2-Fc proteins might allow simultaneous binding of the two ACE2 PDs to two RBDs of a single S-protein trimer, which might enhance the avidity of the drug, and also whether the binding of a second ACE2-Fc to an S-protein trimer might be impaired by steric hindrance from a first ACE2-Fc. In addition to enhancing affinity, ACE2 PDs binding simultaneously to more than one RBD on a single S-protein trimer may destabilize the noncovalent interaction between the S1 and S2 polypeptides of the S protein, increasing the likelihood of the S-protein refolding from the pre- to postfusion state (*40*, *41*). We will discuss the potential implications of this for viral neutralization by ACE2 decoys later in the article.

The PD-CLD-Fc protein comprises both the PD (18 to 615) and the CLD (616 to 740) of the extracellular region of ACE2 fused to the N terminus of a human immunoglobulin G4 (IgG4) Fc domain with S228P and L235E substitutions to inhibit IgG4 Fab-arm exchange and Fc effector functions, respectively (IgG4–SPLE). The PD contains the binding site for the RBD of coronavirus S proteins (*14*, *15*). The CLD is composed of a portion that mediates ACE2 homodimerization and a membrane-proximal portion that is flexible (Fig. 2C) (*42*). While the membrane-proximal portion of the CLD might allow the PDs flexibility with respect to the Fc domain, the movement of the two PDs with respect to one another is likely constrained by the noncovalent homodimerization mediated by the CLD. The relevance of PD orientation is illustrated by comparing the cryo–electron microscopy structures of an ACE2 extracellular domain homodimer with that of two monomeric ACE2 PDs bound to a single S-protein trimer (Fig. 2E). The PDs in an ACE2 homodimer are oriented in an antiparallel fashion with their N termini rotated by approximately 180° with respect to one another (Fig. 2D). In contrast, two monomeric PDs bound to an S-protein trimer are oriented with their N termini pointing toward one another (Fig. 2F). Because of this, CLD-mediated homodimerization in a single PD-CLD-Fc molecule may prevent its two PDs from simultaneously binding two RBDs on an S-protein trimer.

However, ACE2 homodimerization may enhance the ability of multiple ACE2-Fc molecules to simultaneously bind a single S-protein trimer by minimizing steric hindrance from the unbound PD of each ACE2-Fc. This is because the PD of an ACE2 homodimer that is not bound to the S-protein RBD would be held in close spatial proximity to the bound PD and oriented away from other ACE2 homodimers that are bound to other RBDs in that S-protein trimer. Figure 2G illustrates the predicted structure of three ACE2 homodimers binding simultaneously to a single S-protein trimer generated by superimposing one PD from the structure of the ACE2 homodimer [Protein Data Bank (PDB): 6M18] onto each of the three RBD-bound PDs in a structure of three ACE2 PDs bound to a single S-protein trimer (PDB: 7KJ3). Alignment was performed by minimizing the root mean square deviation (RMSD) between corresponding PD residues. This analysis indicates that the unbound ACE2 PDs are positioned orthogonally with respect to the S-protein trimer and far from other ACE2 homodimers (Fig. 2H), minimizing the potential for steric hindrance. Notably, when three ACE2 homodimers are bound to a single S protein, the ACE2 transmembrane domain α helices appear to align in the same plane (Fig. 2G), which suggests that the S protein may have evolved to bind simultaneously to multiple ACE2 homodimers on the cell surface. We therefore hypothesized that, by mimicking the natural structure of the ACE2 homodimer, PD-CLD-Fc would have a high affinity for the S-protein trimer and potently neutralize virus.

In PD-Fc, we fused the PD of ACE2 to an IgG4-SPLE Fc domain, omitting the CLD. Comparison of PD-Fc with PD-CLD-Fc would allow us to assess the functional effect of the CLD. The smaller size of PD-Fc might afford it better tissue penetration, and the absence of the CLD might allow more movement of the PDs with respect to one another. However, there is reduced spacing and flexibility between the PDs and the Fc domain, owing to the absence of the flexible portion of the CLD and the relatively inflexible hinge region of the IgG4-SPLE Fc (Fig. 2C). In PD-Li-Fc, to increase the spacing and flexibility between the PD and Fc domain, we included a flexible G_4_AG_4_ linker between the PD and hinge of IgG4-SPLE Fc (*43*, *44*). We reasoned that comparison of PD-Li-Fc with PD-Fc could offer insight into the effect of hinge flexibility and domain spacing on binding affinity and viral neutralization.

Last, in Fc-Li-PD, the orientation of the PD and Fc domains is reversed, with the IgG1 Fc domain with L234A and L235A substitutions to inhibit Fc effector functions being followed by a G_4_AG_4_ linker and the PD domain. This design was prompted by our analysis of cryo–electron microscopy structures, which indicate that the distance between the C termini of two ACE2 PDs binding to two RBDs on a single SARS-CoV-2 S-protein trimer is twice the distance between their N termini (Fig. 2E) (*22*). By fusing the PD to the C terminus of the Fc domain, the distance that must be spanned by the linker to enable simultaneous binding of the two PD to two RBD on an S-protein trimer is halved and, thus, more energetically favorable. Moreover, linkage of the N termini of the ACE2 PDs to the C termini of the Fc domain would be expected to constrain the rotation of the PDs to favor orientations in which their N termini are pointed toward one another, which is the orientation most favorable for simultaneous binding to two RBDs (Fig. 2F). We therefore hypothesized that Fc-Li-PD would bind tightly to the S protein via an avidity effect from the simultaneous binding of its two ACE2 PDs with two RBDs of an S protein.

### The structure of an ACE2 decoy affects its apparent binding affinity to the S-protein trimer

**Fig. 3.**
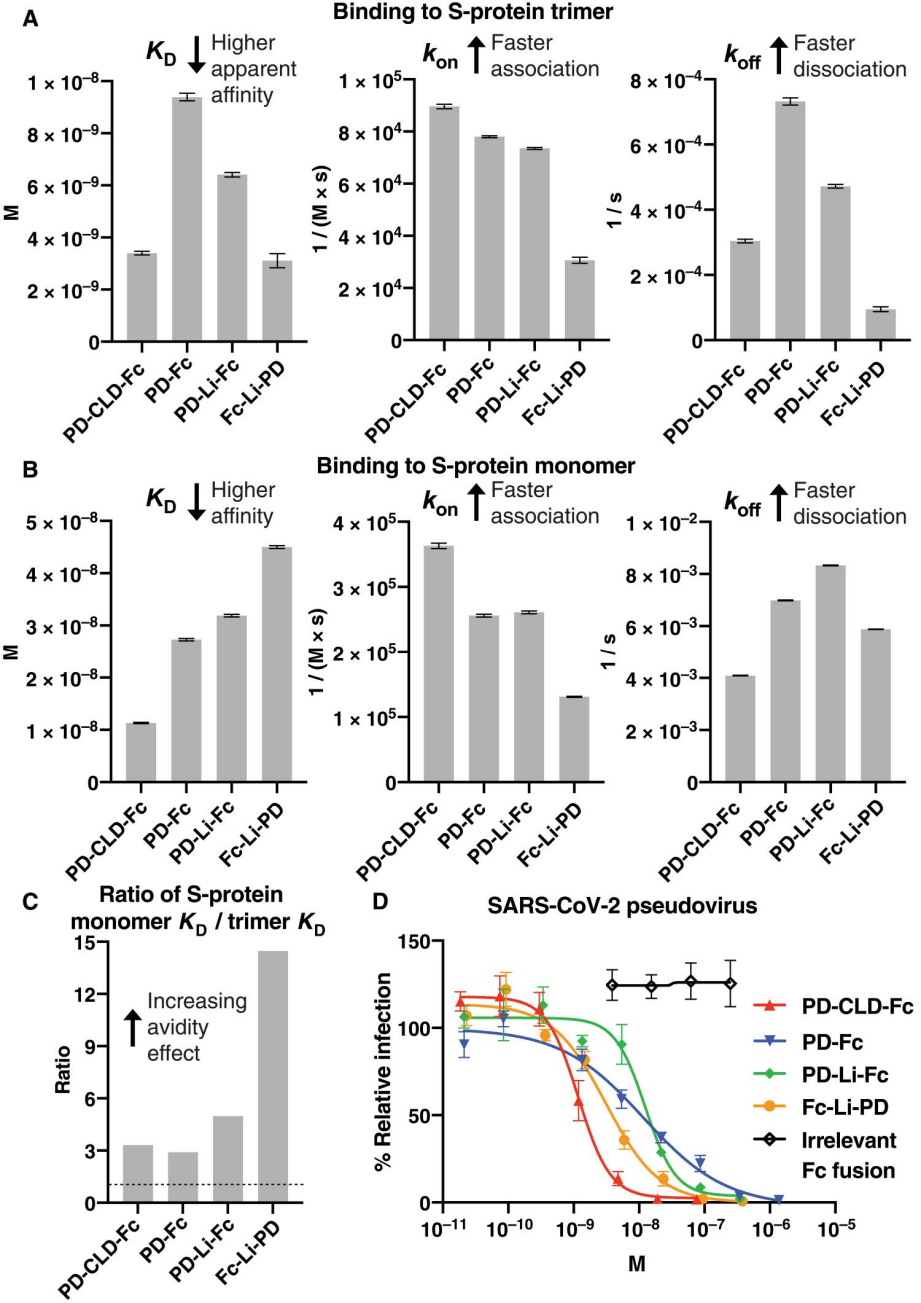
ACE2-Fc structure influences affinity for S protein and the potency of viral neutralization. (**A**) The binding of soluble ACE2-Fc decoys to immobilized prefusion stabilized S-protein trimer was measured by biolayer interferometry (BLI). (**B**) The binding of soluble S1 monomer to immobilized ACE2-Fc decoys was measured by BLI. (**C**) Ratio of binding affinity (*K*_D_) to S-protein monomer and S-protein trimer of the four ACE2-Fc decoys. (**D**) Neutralization of SARS-CoV-2 pseudotyped VSV particle infection of ACE2-transduced HEK293T cells.

We compared the apparent binding affinities of the ACE2-Fc compounds to the prefusion stabilized SARS-CoV-2 S-protein trimer using biolayer interferometry (BLI) (Fig. 3A and fig. S4A). The dissociation constant (*K*_D_) for S-protein binding of the four designs varied by approximately threefold. PD-Fc had the least affinity for the S-protein trimer, with a *K*_D_ of 9.4 nM. PD-Li-Fc bound with a slightly higher affinity (*K*_D_ of 6.4 nM), suggesting that increasing the flexibility and spacing between the PD and the Fc domain enhances binding. PD-CLD-Fc and Fc-Li-PD bound most tightly to the S protein with a *K*_D_ of 3.4 and 3.1 nM, respectively. The higher apparent affinity of PD-CLD-Fc relative to the other N-terminal Fc fusions, PD-Fc and PD-Li-Fc, indicates that inclusion of the CLD improves affinity for the S-protein trimer, which is consistent with prior reports (*18*, *19*). The higher apparent affinity of the C-terminal Fc fusion Fc-Li-PD relative to the N-terminal fusions PD-Fc and PD-Li-Fc indicates that reversing the orientation of the PD by linking it to the C terminus of the Fc domain also enhances its apparent affinity for the S-protein trimer.

### Reversing the orientation of the PD and Fc domains improves apparent S-protein affinity by enhancing avidity

To investigate why Fc-Li-PD and PD-CLD-Fc have high apparent affinities for the S-protein trimer, we compared their apparent affinities for the S-protein trimer versus the S1 monomer (Fig. 3B and fig. S4B). The S1 protein is a cleavage product of the S protein, generated predominantly by proprotein convertases such as furin within the Golgi apparatus of virus-producer cells, and contains a single RBD (*33*). By affixing the ACE2-Fc compounds to the BLI sensor and measuring binding to the soluble S1 monomer, the affinity of the compounds for the RBD monomer can be assessed without the contribution of an avidity effect from the multivalency of the S-protein trimer. The affinities of the four ACE2-Fc compounds for the S1 monomer were substantially lower than those for the S-protein trimer, with *K*_D_ ranging from 11 to 45 nM, indicating that an avidity effect contributes to the higher apparent affinity for the S-protein trimer of each of the compounds. By comparing the ratio of the *K*_D_ for monomer versus trimer for each of the ACE2-Fc proteins, the contribution of the avidity effect for S-protein trimer binding can be assessed, with a higher ratio indicating a more pronounced avidity effect (Fig. 3C). This was most pronounced in Fc-Li-PD, with 15-fold enhancement compared with approximately 3-fold for the other compounds. This and the fact that Fc-Li-PD’s high affinity for S-protein trimer appears to be driven primarily by a slow off-rate rather than a fast on-rate (Fig. 3A) are consistent with our hypothesis that Fc-Li-PD can bind simultaneously to two RBDs on an S-protein trimer, enhancing avidity.

### Including the CLD improves apparent S-protein affinity by enhancing intrinsic affinity for the S-protein RBD, not by enhancing avidity

In contrast to Fc-Li-PD, the high apparent affinity of PD-CLD-Fc for the S-protein trimer appears to be the result of an intrinsically higher affinity for the S-protein RBD rather than an enhanced avidity effect. The *K*_D_ of PD-CLD-Fc for S1 monomer binding was the tightest of the four compounds (about fivefold tighter than Fc-Li-PD) (Fig. 3B), while the contribution of the avidity effect to PD-CLD-Fc’s apparent affinity for the S-protein trimer was similar to that of the other N-terminal ACE2-Fc fusions, PD-Fc and PD-Li-Fc, and far less than that of Fc-Li-PD (Fig. 3C). In addition, PD-CLD-Fc had the highest on-rate of the four compounds for both the S1 monomer and the S-protein trimer (Fig. 3B). These results support the conclusion that PD-CLD-Fc’s high affinity for the S-protein trimer is driven by a higher affinity for the RBD and is not due to an enhanced avidity effect from simultaneous binding of two ACE2 PDs to two RBDs on an S-protein trimer. The restrictions on PD orientation and freedom of movement imposed by CLD-mediated homodimerization may be incompatible with simultaneous binding of two ACE2 PDs to two RBDs on a single S-protein trimer, but they appear to result in a molecule with an intrinsically higher affinity for the individual RBDs within an S-protein trimer.

### Inclusion of the ACE2 CLD most potently enhances viral neutralization

We then compared the ability of each ACE2-Fc to inhibit infection of ACE2-transduced human embryonic kidney (HEK) 293T cells by SARS-CoV-2 pseudovirus (Fig. 3D). A luciferase reporter was used to quantify infection. We found that the IC_50_ of the four ACE2-Fc compounds varied by 10-fold. The potency of viral neutralization generally correlated with the affinity of the compound for the S-protein trimer, with the PD-CLD-Fc having the lowest IC_50_ of 1.2 nM and the reversed orientation Fc-Li-PD having the next lowest IC_50_ of 3.1 nM. PD-Fc and PD-Li-Fc each had IC_50_s of approximately 12 nM, 10-fold less effective than PD-CLD-Fc. Thus, the inclusion of the CLD in an N-terminal ACE2-Fc improves the potency of viral neutralization by 10-fold. C-terminal fusion of the ACE2 PD to an Fc domain also improves viral neutralization compared to N-terminal ACE2-Fc fusions containing only the PD but not compared to N-terminal ACE2-Fc fusions containing both PD and CLD. PD-CLD-Fc also potently neutralizes infection by authentic SARS-CoV-2 of human type 2 alveolar cells derived from induced pluripotent stems cells (iAT2 cells) (fig. S5).

### Inclusion of the ACE2 CLD unexpectedly improves serum half-life

**Fig. 4.**
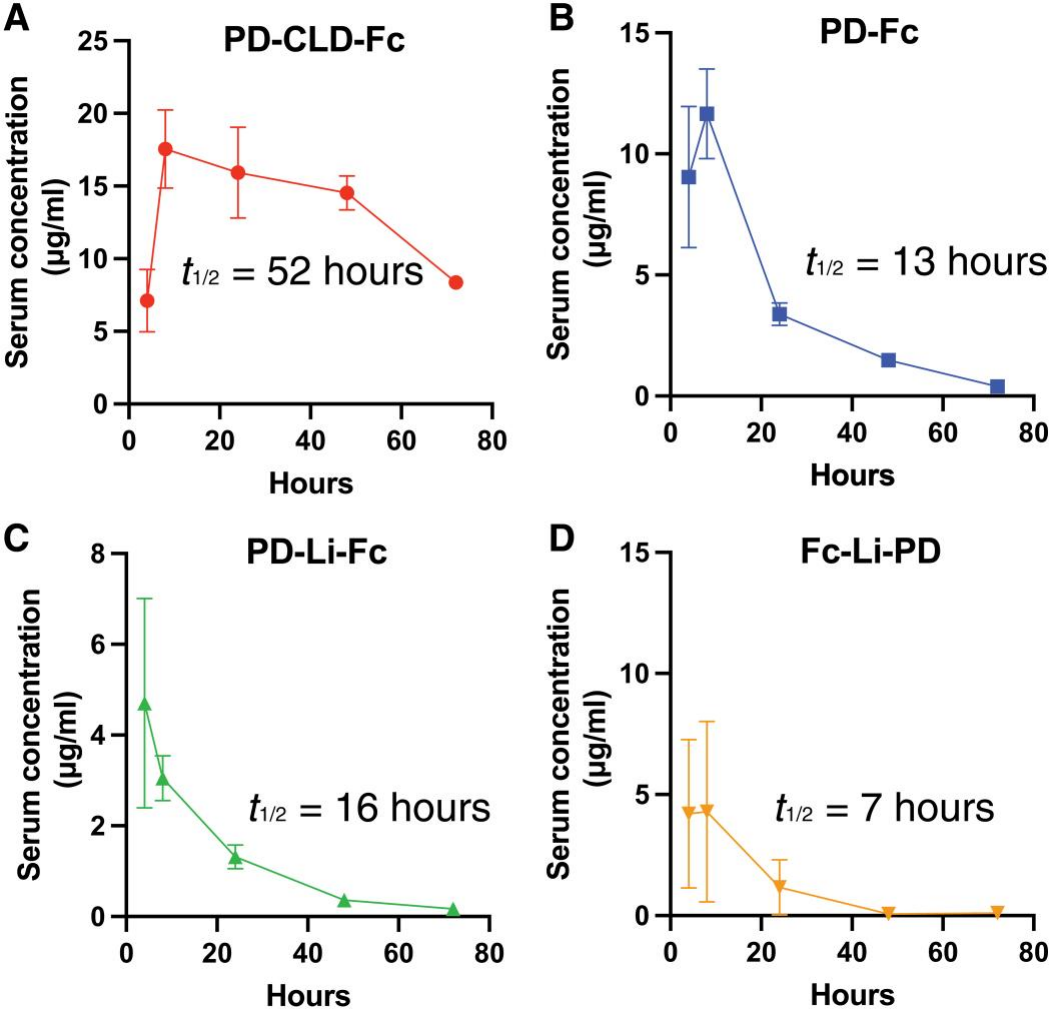
Inclusion of the ACE2 CLD extends serum half-life. Syrian hamsters were injected with 8 mg/kg of (**A**) PD-CLD-Fc, (**B**) PD-Fc, (**C**) PD-Li-Fc, or (**D**) Fc-Li-PD, and serial blood draws were performed. The serum concentration of each compound was assessed by ELISA.

We then compared the serum half-life of the four ACE2-Fc proteins in Syrian hamsters (Fig. 4); 8 mg/kg of each of the compounds was administered via intraperitoneal injection to separate groups of three hamsters at time zero. Venous blood draws were performed at 4, 8, 24, 48, and 72 hours, and the serum concentration of the compounds was determined by enzyme-linked immunosorbent assay (ELISA) for each sample. Unexpectedly, there was a more than sevenfold difference in the serum half-life between the ACE2-Fc compounds. PD-CLD-Fc had by far the longest serum half-life of 51.6 hours. PD-Fc, PD-Li-Fc, and Fc-Li-PD had shorter half-lives of 15.5, 13.0, and 6.7 hours, respectively. To determine whether this was a result of differences in protein stability, susceptibility to proteases, or some other cause, we incubated the compounds in 95% human serum for 48 hours at 37°C. They remained intact, and their affinities for immobilized SARS-CoV-2 S protein were unchanged as measured by ELISA (fig. S6). This argues that the observed differences in serum half-life in hamsters are not due to instability or proteolytic cleavage but instead due to intrinsic differences in the pharmacokinetics of the proteins. Unexpectedly, inclusion of the CLD prolongs the serum half-life of an ACE2-Fc decoy.

### Inclusion of the ACE2 CLD is necessary for activity against SARS-CoV-2 in hamsters

**Fig. 5.**
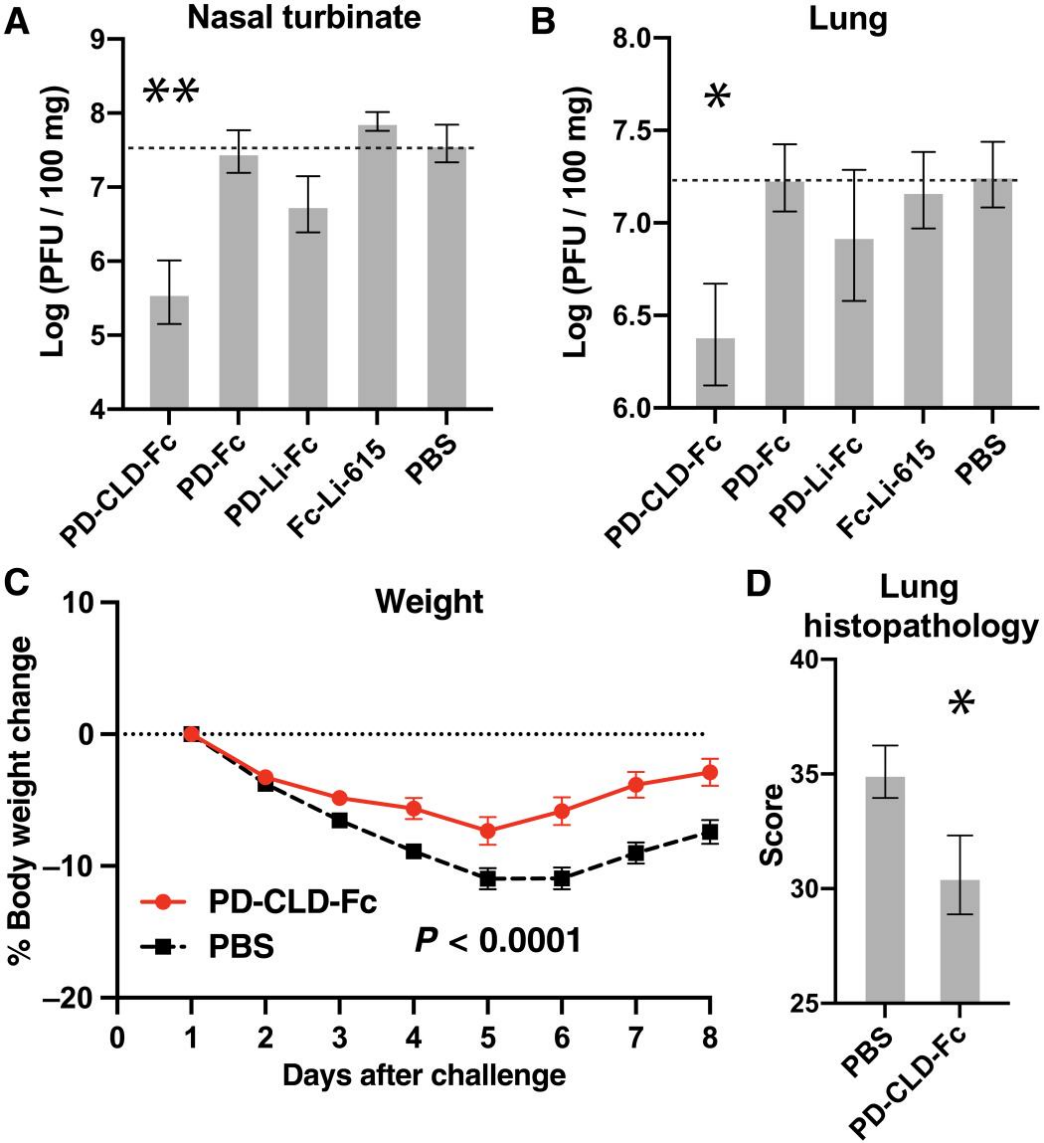
Inclusion of the ACE2 CLD is necessary for in vivo antiviral activity. (**A** and **B**) Syrian hamsters were treated by intraperitoneal injection with either PD-CLD-Fc (40 mg/kg) every 24 hours, PD-Fc (20 mg/kg) every 12 hours, PD-Li-Fc (20 mg/kg) every 12 hours, or Fc-Li-PD (20 mg/kg) every 12 hours beginning 8 hours before intranasal instillation of 10^4^ plaque-forming units (PFU) of SARS-CoV-2 virus. All animals were euthanized on day 3. (A) Nasal turbinate viral titer was measured for each animal at day 3. Statistical significance was evaluated using a two-sided *t* test. ** denotes a *P* value of 0.0024. (B) Cranial and caudal lung titer was measured for each animal at day 3. Statistical significance was evaluated using a two-sided *t* test. * denotes a *P* value of 0.015. (**C** and **D**) To model treatment in the therapy setting, Syrian hamsters were challenged with 10^4^ PFU of SARS-CoV-2 virus by intranasal instillation and then treated 12 hours later with a single intraperitoneal injection of PD-CLD-Fc (150 mg/kg). (C) Body weight change relative to the onset of treatment. Statistical significance was evaluated using a mixed-effects model. (D) Animals were euthanized on day 8. The severity of alveolitis, vasculitis, lesion extent, pneumocyte hyperplasia, interstitial inflammation, and bronchitis was scored, and a composite average was calculated for each lung and then averaged for each animal. Statistical significance was evaluated using a two-sided *t* test. * denotes a *P* value of 0.043.

We compared the in vivo antiviral activities of the four ACE2-Fc compounds in a rodent model of SARS-CoV-2 infection. When challenged intranasally with SARS-CoV-2, Syrian hamsters demonstrate rapid weight loss, high viral titers in nasal turbinates and lung, and severe lung pathology (*45*, *46*). We compared the ability of the four ACE2-Fc proteins to alter the disease course in this model by administering a total of 40 mg/kg per day of ACE2-Fc or vehicle [phosphate-buffered saline (PBS)] for 3 days beginning 8 hours before intranasal challenge with 1 × 10^4^ plaque-forming units (PFU) of the WA01 isolate of SARS-CoV-2. All animals were euthanized on day 3. We determined body weight loss and obtained viral titers from homogenates of the nasal turbinates and lungs. PD-CLD-Fc was the only compound to attenuate disease severity as measured by weight loss (fig. S7). Treatment with PD-CLD-Fc resulted in a statistically significant reduction of approximately 100-fold in nasal turbinate viral titers (Fig. 5A) and 10-fold in combined cranial and caudal lung titers (Fig. 5B). None of the other compounds resulted in a statistically significant reduction in nasal turbinate or lung viral titers.

We therefore selected PD-CLD-Fc as our lead compound since it inhibited viral infection most potently in vitro, had the longest half-life in hamsters, and was the only compound to reduce nasal turbinate and lung viral titer in a hamster model of SARS-CoV-2 infection. We evaluated the ability of PD-CLD-Fc to treat hamsters in a therapeutic model of SARS-CoV-2 infection, in which hamsters are challenged with virus and, after a delay, treated with drug. A total of 40 hamsters were challenged by intranasal instillation with 1 × 10^4^ PFU of SARS-CoV-2. Twelve hours later, half of the hamsters (20) were treated with a single dose of PD-CLD-Fc (150 mg/kg) administered by intraperitoneal injection, and the other half (20) were treated with an intraperitoneal injection of vehicle (PBS). Daily weights were recorded for each animal. A cohort of 10 hamsters from the PD-CLD-Fc group and 10 hamsters from the PBS group was euthanized on day 8, and lung tissue was fixed and sectioned for histopathological analysis. Another cohort from each group was euthanized on day 4 to obtain viral titers in nasal turbinate and lung.

Treatment with PD-CLD-Fc resulted in a highly statistically significant reduction in weight loss compared with placebo, indicating that PD-CLD-Fc can attenuate the severity of disease when administered in the therapeutic setting (Fig. 5C). Treatment with PD-CLD-Fc also resulted in a statistically significant reduction in the severity of lung pathology as measured by a composite index of alveolitis, pneumocyte hyperplasia, vasculitis, interstitial hyperplasia, lesion extent, and bronchitis (Fig. 5D). Despite the apparent improvement in disease severity and improvement in lung histopathology, there was no statistically significant reduction in nasal turbinate or lung viral titer seen on day 4 (table S1).

### ACE2 decoys may inhibit viral infection by triggering refolding of the S protein, enhancing their potency

**Fig. 6.**
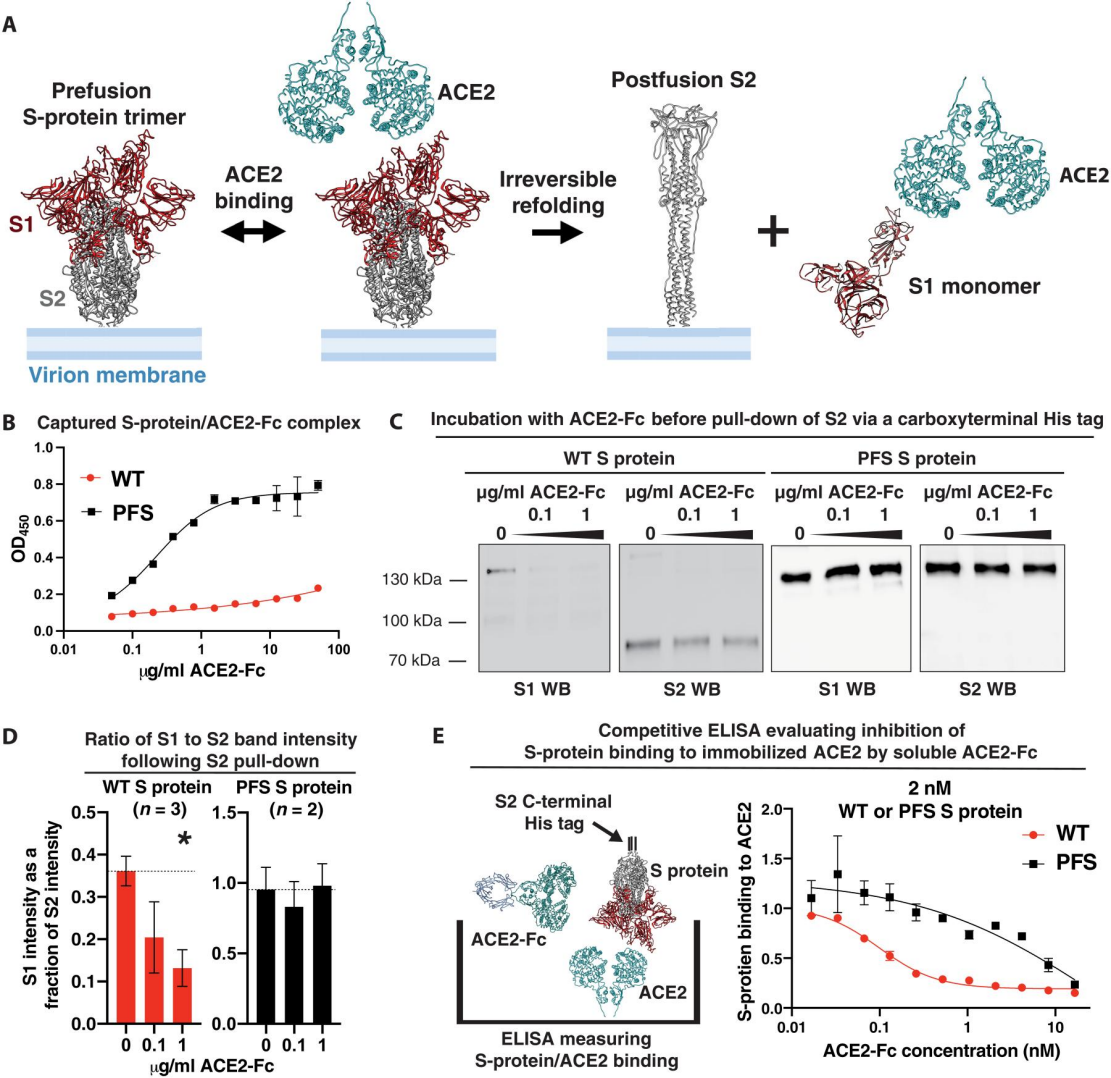
ACE2-Fc binding triggers refolding and dissociation of the S-protein complex, enhancing the potency of ACE2 binding inhibition. (**A**) Binding of viral S-protein RBD to cell-surface ACE2 is known to trigger a structural change in S protein from a prefusion state in which S1 and S2 polypeptides are noncovalently associated to a postfusion state in which S1 polypeptide dissociates from S2, allowing S2 polypeptide to refold and extend. The postfusion S2 polypeptide remains anchored to the virion membrane but, having lost the RBD-containing S1, is no longer capable of binding ACE2. (**B** to **E**) To evaluate whether ACE2-Fc in solution triggers this refolding, recombinant soluble wild-type (WT) or prefusion stabilized (PFS) S-protein trimer was incubated with varying concentrations of PD-CLD-Fc for 15-min in the presence of trypsin to mimic S1/S2 cleavage by furin and TMPRSS2. (B) The PD-CLD-Fc/S-protein complex was captured using plate-bound anti-S2 monoclonal antibody and detected using horseradish peroxidase (HRP)–conjugated antihuman IgG antibody. (C) His-tagged S2 on WT and PFS S proteins was pulled down using nickel beads. S2 or coprecipitating S1 was detected by Western blot using S1- or S2-specific monoclonal antibody. (D) The ratio of S1 to S2 band intensity (S1/S2) was calculated for each experimental condition across three experiments with WT S protein and two experiments with PFS S protein. * denotes a *P* value of 0.015. (E) To evaluate the importance of refolding for decoy-mediated ACE2 binding inhibition, WT or PFS S proteins (with C-terminal His tags) were preincubated with PD-CLD-Fc as above and transferred to an ELISA plate coated with ACE2. S-protein binding was detected with HRP-conjugated anti–His tag antibody and normalized to samples with no PD-CLD-Fc added.

ACE2-Fc decoys are presumed to neutralize virus by competitively inhibiting the binding of the viral S protein to cell-surface ACE2. Binding of cell-surface ACE2 to the S protein is known, however, to trigger a structural change in the S protein from a “prefusion” state in which the S1 and S2 polypeptides are noncovalently associated to a “postfusion” state in which the S1 polypeptide dissociates from S2, allowing the S2 polypeptide to refold and adopt an extended conformation (Fig. 6A) (*33*, *34*). S2 remains embedded in the membrane of the virion, and this refolding event is thought to promote fusion of the virion membrane with the host-cell plasma membrane (*33*, *34*). Since the S2 polypeptide does not contain the RBD, the refolded postfusion S protein is no longer capable of binding to ACE2. In light of reports that recombinant soluble ACE2 PD can mimic cell-surface ACE2 and trigger the refolding of SARS-CoV S-protein trimer from a pre- to postfusion state (*35*), we considered the possibility that ACE2-Fc decoys may function similarly and that mimicry of this receptor activity might contribute to the ability of ACE2-Fc to neutralize infectivity.

To address this hypothesis, we evaluated the ability of PD-CLD-Fc to facilitate the dissociation of S1 and S2 polypeptides in recombinant S-protein trimer, an event that occurs when the S protein refolds during infection. We treated S-protein trimer with varying concentrations of PD-CLD-Fc in the presence of trypsin to ensure cleavage of S protein into S1 and S2 polypeptides (*35*, *47*, *48*). Treated samples were analyzed using an ELISA in which the S protein was captured by plate-bound antibody specific for the S2 polypeptide, and association of the PD-CLD-Fc with the captured S protein was detected using antihuman IgG–horseradish peroxidase (HRP). Since PD-CLD-Fc binds the RBD on the S1 polypeptide, association of the PD-CLD-Fc compound with the S2 polypeptide should be detected if the S1 and S2 polypeptides remain associated with one another but not if the S2 protein has refolded and the S1 has dissociated. A concentration-dependent increase in PD-CLD-Fc binding was observed using prefusion stabilized (PFS) S protein in which the furin cleavage site between the S1 and S2 polypeptides has been removed, and amino acid substitutions were added to inhibit the refolding of S2 to the postfusion state (Fig. 6B). In contrast, no association of PD-CLD-Fc with wild-type (WT) S protein was detected, which we hypothesized was due to PD-CLD-Fc–mediated refolding of the WT S protein and dissociation of the S1 polypeptide from S2 (Fig. 6B). To directly assess the association of the S1 polypeptide with the S2 polypeptide, we used a coprecipitation assay, in which the S2 polypeptide was pulled down via a C-terminal His tag, and the associated S1 was detected by an anti-S1 Western blot (Fig. 6C). In both WT and PFS S protein, S1 coprecipitated with S2 in the absence of the PD-CLD-Fc compound. As WT S protein was exposed to increasing concentrations of PD-CLD-Fc, less S1 coprecipitated with S2, indicating that PD-CLD-Fc triggered dissociation of S1 from S2. In contrast, when PFS S protein was exposed to increasing concentrations of PD-CLD-Fc, the amount of S1 coprecipitating with S2 remained constant, consistent with the inability of S1 to dissociate from S2 in the PFS S protein. This was confirmed quantitatively across independent experiments by calculating the ratio between the S1 and S2 signals measured by densitometry (Fig. 6D). These findings demonstrate that ACE2 decoys can mimic cell-surface ACE2 and trigger dissociation of S1 from S2, resulting in a postfusion S2 polypeptide on the virion membrane that is no longer capable of binding cell-surface ACE2.

We then compared the ability of PD-CLD-Fc to inhibit the binding of WT or PFS S-protein trimer to immobilized ACE2. We found that PD-CLD-Fc potently inhibited the binding of the WT S protein to ACE2 but was much less effective at inhibiting binding of PFS S protein (Fig. 6E). An equimolar amount (2.2 nM) of PD-CLD-Fc was capable of nearly maximal inhibition of the WT S protein, but it had minimal effect on the PFS S protein. PD-CLD-Fc inhibited binding of WT S protein to immobilized ACE2 with an IC_50_ of approximately 0.1 nM but inhibited binding of PFS S protein far less potently with an IC_50_ of approximately 10 nM (Fig. 6E). Thus, the inhibitory effect of PD-CLD-Fc is far more potent on a natural S protein capable of refolding to the postfusion state than on an S protein that remains in the prefusion state, suggesting that S-protein refolding is important for the mechanism of ACE2 decoys.

**Fig. 7.**
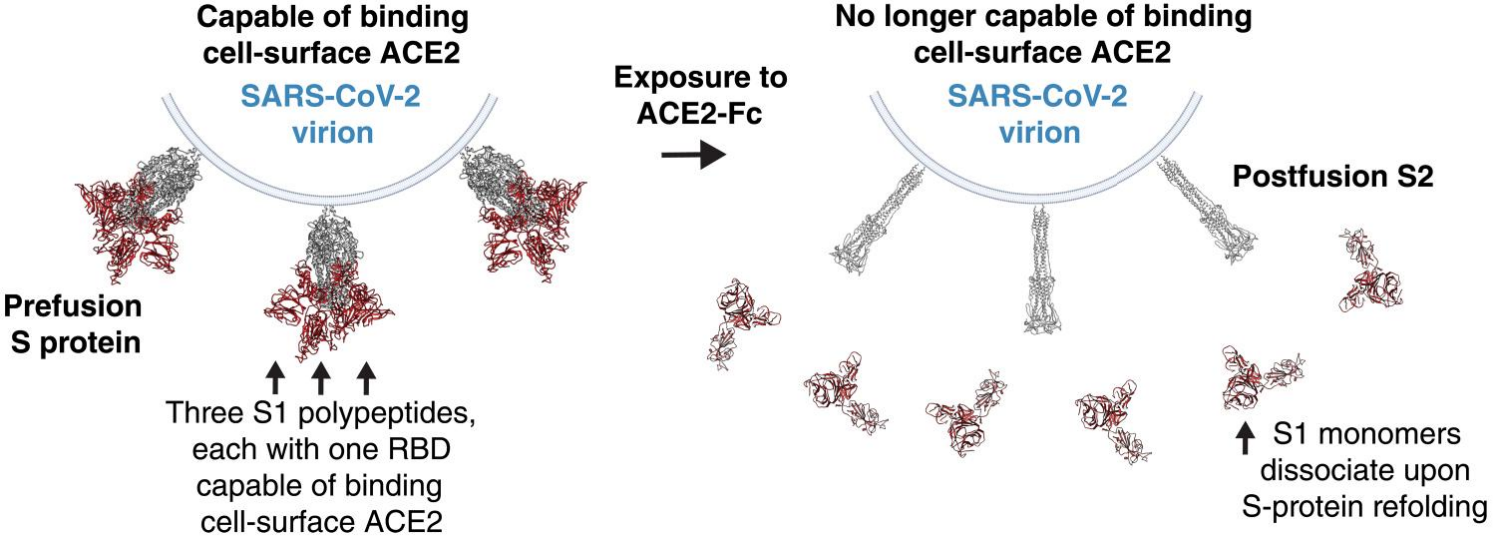
Proposed mechanism of ACE2-Fc decoy–mediated virion inactivation through the premature triggering of S-protein refolding via receptor mimicry. Binding of the decoy triggers irreversible refolding of viral S proteins from the pre- to the postfusion state. Having lost the RBD-containing S1 polypeptide, the postfusion S2 on the virion surface is no longer capable of binding ACE2 on the target cell surface, which is necessary for infection.

The fact that ACE2-Fc decoys trigger conformational change of the S protein may help to explain their potent inhibitory effect against coronavirus. Since the postfusion S protein is incapable of binding cell-surface ACE2, an ACE2-Fc decoy does not have to compete continuously with cell-surface ACE2 for binding to the S-protein RBD to inhibit viral infection. Rather, engagement of the S protein by the ACE2-Fc decoy can render a viral S protein “inactive” by binding it and facilitating its transition to the postfusion state (Fig. 7). Moreover, since the off-rate for ACE2-Fc from the S1 monomer was more than 10-fold faster than from the S-protein trimer (Fig. 2, A and B), it is possible that an ACE2-Fc molecule might participate in the inactivation of additional S-protein trimers after dissociating from an S1 monomer.

## DISCUSSION

In this study, we find that the activity of ACE2-Fc decoys is not reduced by naturally selected mutations in its ligand, SARS-CoV-2 S protein, which imparts resistance to anti–S protein antibodies. An ACE2-Fc decoy maintained and, in fact, gained potency against the Omicron BA.1 variant, which has 30 amino acid changes in the S protein, making it resistant to neutralization by almost all therapeutic monoclonal antibodies that have been developed to treat COVID-19 (*2*). The ACE2-Fc also neutralized the Beta variant, another antibody-resistant variant, without any loss in potency, and it binds tightly to the S1 proteins from the Alpha, Beta, Gamma, Delta, Epsilon, and Omicron BA.1 variants.

To optimize the design of an ACE2 decoy therapeutic, we compare ACE2-Fc designs to identify elements that improve S-protein binding and viral neutralization but do not involve mutation of ACE2 surface residues. This reduces the likelihood of anti-ACE2 antibodies developing that might impair drug activity and cross-react with endogenous cell-surface ACE2. It also avoids overcustomization of the decoy to a specific coronavirus S protein, which might undermine activity against future SARS-CoV-2 variants and ACE2-dependent coronaviruses.

We find that inclusion of the ACE2 CLD in addition to the ACE2 PD improves apparent affinity for the viral S protein, as has been previously reported (*18*, *19*, *23*). We also find that a C-terminal Fc-ACE2 fusion with reversed domain orientation (Fc-Li-PD) has a similarly high apparent affinity. However, we find that the reason for the high apparent affinity of these two designs is different: Reversing the orientation of the PD and the Fc domain enhances avidity for the S protein, whereas including the CLD improves intrinsic affinity for the RBD but does not enhance avidity for the S protein. Findings from our structural analysis are consistent with our experimental findings: The orientation and distance between ACE2 PDs of a C-terminal Fc-ACE2 fusion should allow them to bind simultaneously to two different RBDs on a single S-protein molecule, whereas, in a CLD-containing ACE2-Fc, the PDs are fixed in an orientation and at a distance that is incompatible with simultaneous binding to two RBDs on an S protein. Instead, CLD-mediated homodimerization appears to minimize steric hindrance by the unbound PD, allowing multiple ACE2-Fc (up to three) to bind to a single S protein.

The potency of viral neutralization correlates generally with apparent S-protein affinity. However, among ACE2 decoys with a similar apparent S-protein affinity, a faster on-rate yields more potent viral neutralization than a slower off-rate. The CLD-containing PD-CLD-Fc and the reversed orientation Fc-Li-PD had the highest apparent S-protein affinities and also neutralized SARS-CoV-2 pseudovirus more potently than ACE2-Fc decoys of other designs. However, PD-CLD-Fc, which has a faster on-rate, neutralized virus twofold more potently than Fc-Li-PD, which has a slower off-rate. Thus, the rate of RBD engagement may matter more than the persistence of RBD engagement.

Unexpectedly, we find that inclusion of the ACE2 CLD also prolongs serum half-life of an ACE2-Fc by four- to sevenfold. As an apparent consequence, the CLD-containing ACE2-Fc (PD-CLD-Fc) was the only compound capable of attenuating weight loss and reducing viral titer in a hamster model of SARS-CoV-2 infection. PD-CLD-Fc also attenuated weight loss and improved lung pathology when administered therapeutically as a single dose 12 hours after viral challenge. While we show a statistically significant viral reduction in the prophylactic setting, the failure to reduce viral titer in the therapeutic setting could be viewed as a limitation. However, no statistically significant decrease in lung viral titer in hamsters was seen in either the prophylactic or therapeutic setting in a published study of casirivimab/imdevimab antibody cocktail (*49*). Moreover, the attenuation of weight loss in hamsters treated with PD-CLD-Fc in our study was comparable to that seen in casirivimab/imdevimab-treated hamsters (*49*). That PD-CLD-Fc exhibits comparable efficacy in hamsters to a clinically effective antibody cocktail bodes well for its likelihood of achieving a similar therapeutic effect in humans but with the added benefit of lasting efficacy in the context of a constantly changing virus.

In addition, we find that ACE2-Fc decoys work by functionally mimicking cell-surface ACE2 to trigger refolding of the S protein from the pre- to postfusion state, resulting in dissociation of the S1 polypeptide from the S2 polypeptide. When the S protein refolds, the postfusion S2 remains on the surface of the virion but does not contain an RBD; therefore, it is no longer capable of binding cell-surface ACE2 and mediating infection. This “inactivation” of the viral S protein by refolding could explain why ACE2 decoys potently neutralize SARS-CoV-2. ACE2-Fc inhibits the binding of WT S protein to immobilized ACE2 far more potently than it inhibits binding of S protein stabilized in the prefusion state, consistent with the hypothesis that ACE2 decoys inhibit viral infection not only through competitive inhibition of the S protein–ACE2 interaction but also by triggering the irreversible refolding of the S protein from the prefusion state to the postfusion state. When more than one RBD on an S-protein trimer binds an ACE2 PD, the likelihood of S protein refolding from the pre- to postfusion state is increased (*40*, *41*). Our structural analysis suggests that three ACE2-Fc molecules can bind to three RBDs on a single S-protein trimer without steric hindrance, and this could be one explanation for the superior neutralization potency of the CLD-containing ACE2-Fc. Furthermore, an ACE2-Fc decoy that binds more quickly to the S protein would be expected to neutralize S protein more potently than one that binds more slowly but remains bound for longer. This could explain why the ACE2-Fc decoy with the highest on-rate, PD-CLD-Fc, neutralizes virus more potently than the other decoys, including Fc-Li-PD, which has a similar apparent affinity but a slower on-rate and off-rate.

A consideration in the design of ACE2-Fc soluble decoy receptors is whether to silence the enzymatic activity of the ACE2 PD. Although it has been postulated that enzymatically active ACE2 might treat or prevent acute respiratory distress syndrome (ARDS) in patients with COVID-19, largely based on the preclinical finding that soluble ACE2 protects against ARDS in a murine acid aspiration model (*50*), results from two phase 2 clinical trials have failed to demonstrate a clinical benefit in patients. A phase 2 trial of enzymatically active ACE2 in patients with ARDS, performed before the pandemic, failed to show a benefit in lung compliance, lung oxygenation, or Sequential Organ Failure Assessment score (*51*). This failure to demonstrate a clinical benefit was despite a successful reduction of serum angiotensin II levels and an increase in serum angiotensin (1 to 7) levels, the hypothesized mechanisms of benefit. More recently, a randomized placebo–controlled phase 2 clinical trial of enzymatically active ACE2 in 181 patients hospitalized with COVID-19 also failed to demonstrate a clinical benefit (*52*). Since ACE2 inactivates angiotensin II and inhibits the RAAS, an important regulator of blood pressure, administration of exogenous enzymatically active ACE2 in doses high enough to inhibit virus could result in hypotension, particularly in individuals in whom maintenance of blood pressure is reliant on RAAS activation, such as those in states of intravascular volume depletion or in chronic states of RAAS activation due to renal artery stenosis, heart failure, and cirrhosis. These physiologic states are more common in populations at increased risk for severe COVID-19. Although enzymatically active ACE2 was safe when administered at 0.4 and 0.8 mg/kg in the trials above, the dose of an ACE2-Fc decoy required for antiviral effect is anticipated to be on the order of 100-fold higher than this. Therefore, we believe that silencing the ACE2 enzymatic activity of an ACE2 decoy to isolate its antiviral activity and widen its therapeutic index is prudent and may be required to safely achieve doses sufficient for antiviral effect.

Last, silencing the Fc effector functions of an ACE2-Fc decoy may mitigate the risk of immunopathologic adverse effects and allow it to be used safely in patients with severe COVID-19. Therapeutic antibody use is contraindicated in these patients because of concern that a subset of these patients may be harmed, and FDA Emergency Use Authorizations for therapeutic antibodies explicitly exclude hospitalized patients or those who require oxygen therapy because of COVID-19 (*53*, *54*). An industry-sponsored trial of casirivimab/imdevimab was stopped early in hospitalized patients on high-flow oxygen due to the concern for harm (*55*). Results from the RECOVERY trial, in which hospitalized COVID-19 patients with severe disease were treated with casirivimab/imdevimab, suggest a trend toward worsened mortality in seropositive patients (those who had an endogenous SARS-CoV-2 antibody response) but indicate improved mortality in seronegative patients (those without an endogenous antibody response) (*56*). We speculate that harm from monoclonal antibody therapy in the seropositive subset might be due to FcγR-mediated ADE of inflammation driving the severe COVID-19 disease process in these patients. This is supported by accumulating evidence that the Fc effector functions of antibodies can promote immunopathology. Patients with severe COVID-19 have a higher proportion of afucosylated anti-RBD antibodies, which bind FcγRs more avidly, and viral immune complexes containing these antibodies triggered more potent innate immune cell activation and release of proinflammatory cytokines, such as interleukin-6 (IL-6), IL-1β, and tumor necrosis factor (*57*, *58*). Circulating monocytes in patients with COVID-19 are infected with the SARS-CoV-2 virus, and this infection is dependent on FcγR-mediated uptake of antibody-opsonized virus (*39*). Although not productive of virus, this infection of monocytes results in inflammasome assembly and triggers inflammatory cell death (pyroptosis), processes that result in inflammatory cytokine release and may promote the immunopathology of severe COVID-19 (*39*). Monoclonal antibodies can similarly mediate the entry of pseudotyped coronavirus into FcγR-expressing cells and trigger the release of proinflammatory cytokines that exacerbate severe COVID-19 (*37*). Thus, therapeutics with intact Fc effector functionality might paradoxically worsen immunopathology when administered to patients with severe COVID-19. Integrating these findings with those of preclinical studies that demonstrate that enhancing Fc effector functionality improves viral clearance (*59*–*61*), one might conclude that Fc effector functionality is beneficial in earlier stages of coronavirus infection but contributes to the immunopathology of later-stage severe disease. Since our findings indicate that an ACE2-Fc without Fc effector functionality can attenuate disease severity and improve lung pathology in hamsters, in agreement with a published study (*26*), ACE2-Fc decoys with silenced Fc effector functionality might be effective and safer for the treatment of severe COVID-19, allowing their use in hospitalized patients in whom monoclonal antibody use is contraindicated because of the potential for harm. It merits investigation whether such an ACE2-Fc would also reduce immunopathology driven by immune complexes of virus with endogenous antibodies by competing with antibodies that bind the RBD and by disrupting the binding of other S protein–directed antibodies through ACE2 decoy–mediated refolding and disassembly of the S protein.

The selective pressure placed upon SARS-CoV-2 to evade humoral immunity has resulted in variants with mutations in the S protein that escape endogenous antibody binding but retain binding to ACE2 for infectivity. This also allows escape from neutralizing monoclonal antibodies, limiting the useful lifespan of antibodies as therapeutics. In contrast, selection for infectious and fit virions has favored that the S protein of SARS-CoV-2 maintain binding to ACE2. Therefore, the effectiveness of ACE2-Fc decoys should not be susceptible to naturally selected mutations in the S protein, and they could represent lasting therapeutics as SARS-CoV-2 becomes endemic in the human population.

## MATERIALS AND METHODS

### Structural analysis

Structures were downloaded from the PDB, and analysis was performed using UCSF Chimera (*62*). Three-dimensional alignment by RMSD minimization was performed with MatchMaker using the Needleman-Wunsch algorithm (*63*).

### Proteins

The sequences of human ACE2, human IgG1, and human IgG4 were obtained from the Universal Protein Resource (UniProt IDs: Q9BYF1, P01857, and P01861). In all constructs, the point substitutions H374N and H378N were introduced in the ACE2 sequence to render it catalytically inactive. For constructs with an IgG1 Fc domain, L234A and L235A substitutions were introduced to attenuate FcγR binding. For constructs with an IgG4 Fc domain, S228P and L235E substitutions were included to inhibit IgG4 Fab-arm exchange and Fc effector functions, respectively. Gene synthesis and protein production were performed by a contractor (LakePharma). In brief, codon-optimized nucleotide sequences were synthesized and cloned into the pLEV123 expression vector. Chinese hamster ovary cells were transiently transfected with this expression vector and maintained as a batch-fed culture in serum-free chemically defined medium for 14 days. Conditioned medium was harvested and clarified by centrifugation and filtration and ACE2-Fc fusion proteins were purified by a protein A column. Capillary electrophoresis analysis of the target protein was performed using LabChip GXII (PerkinElmer). To confirm a low endotoxin level, duplicate samples of the purified product were quantified using the chromogenic Limulus Amebocyte Lysate method (Endosafe-MCS, Charles River Laboratories). To determine whether aggregates were present in the purified protein product, size-exclusion ultra performance liquid chromatography (SE-UPLC) analysis was performed using an ACQUITY UPLC Protein BEH SEC 200 using a 1.7-μm, 4.6 × 150 mm column (Waters) and a mobile phase of 50 mM sodium phosphate and 500 mM sodium chloride at pH 6.2. Protein products with an aggregate fraction of >10% were polished via size exclusion chromatography using a HiLoad 26/600 Superdex 200 column with a mobile phase of PBS at pH 7.4. Collected and pooled fractions were once again assayed by SE-UPLC to ensure >90% purity. Pooled fractions were passed through a 0.2-μm membrane filter.

### Biolayer interferometry

Binding assays were conducted using the Octet RED384 system (Sartorius). To measure binding to the S-protein trimer, a purified recombinant prefusion-stabilized SARS-CoV-2 S-protein trimer (LakePharma) was immobilized to Amine Reactive 2nd-Generation (AR2G) biosensors (Sartorius) via the manufacturer’s recommended protocol. These coated biosensors were then exposed to solutions of ACE2-Fc proteins at concentrations ranging from 0.39 to 100 nM diluted in running buffer consisting of PBS and 0.02% Tween 20 with bovine serum albumin (BSA, 2 mg/ml) in a 96-well plate. After 5 min of measuring association, biosensors were transferred to wells with running buffer alone for 10 min to measure the dissociation. Reference signal from sensors with no S protein immobilized exposed to solutions of ACE2-Fc or running buffer was subtracted.

To measure binding to S1 proteins from SARS-CoV-2 D614G parent and variants, the ACE2-Fc proteins were immobilized to antihuman IgG Fc capture biosensors (Sartorius) following the protocol recommended by the manufacturer. The S1 proteins (Sino Biological) were diluted in running buffer [PBS, 0.02% Tween 20, and BSA (2 mg/ml)] to 1.23 to 100 nM and transferred to the 96-well plate. The sensors immobilized with the ACE2 variants were dipped in the wells of the S1 proteins for 5 min to measure the association step, followed by dipping in the running buffer for 10 min to measure the dissociation step. The reference signal from sensors with no ACE2-Fc immobilized exposed to solutions of S1 protein or running buffer was subtracted.

Kinetic analysis was performed using the software Octet Data Analysis HT version 12.0. The binding curves of the soluble S trimer binding with ACE2 variants were fit to bivalent mode. The curves of the S1 proteins binding with the ACE2 variants were fit to 1:1 binding mode.

### SARS-CoV-2 pseudoparticle inhibition assays

HEK293T cells overexpressing ACE2 were seeded in poly-l-lysine–coated 96-well plates at a density of 1.5 × 10^4^ cells per well and incubated overnight at 37°C. The next day, SARS-CoV-2/vesicular stomatitis virus (VSV) pseudoparticles were preincubated with different concentrations of ACE2-Fc proteins, PD-L1-Fc (an irrelevant Fc fusion protein as a negative control), or SARS-CoV-2 spike–neutralizing antibody (Sino Biological; 40592-MM57) for 1 hour at 37°C and added to the cells. Twenty-four hours later, the cells were harvested for the luciferase assay. To harvest the cells, the floating cells resulting from the cytopathic effects of VSV replication were collected by transferring the culture medium of all wells to a round-bottom 96-well plate and centrifuging the plate at 1000*g* for 10 min at 4°C. The remaining cells in the monolayer were lysed in 50 μl of 1× FLuc-lysis buffer (GeneCopoeia), and the lysates were transferred to the cells recovered from the culture medium. Twenty microliters of these lysates were then subjected to the luciferase assay using the Luc-Pair Firefly Luciferase HS Assay Kit (GeneCopoeia; LF009). The luciferase activity was measured using a VarioSkan LUX plate reader (Thermo Fisher Scientific), and the neutralization efficiency was determined relative to the no-compound control.

For flow cytometry inhibition assays, A549 cells expressing human ACE2 with a C-terminal hemagglutinin-FLAG tag (BEI NR-53522) were cultured in Dulbecco’s modified Eagle’s medium (DMEM) containing 10% fetal bovine serum (FBS) and 1% penicillin-streptomycin (100 U/100 μg final concentration). To enhance ACE2 expression, cells were passaged twice in media containing puromycin antibiotic (first passage, 0.5 μg/ml; second passage, 1 μg/ml), and the resulting cells were used for subsequent experiments. Cells were seeded in 96-well flat-bottomed cell culture plates at a density of 4 × 10^4^ cells per well and incubated overnight at 37°C 5% CO_2_. The next day, ultracentrifuge-purified VSV pseudovirus particles encoding Zs-Green and incorporating either SARS-CoV-2 S-protein D614G or Beta variant (generated using BEI NR-53817) were preincubated at indicated concentrations of PD-CLD-Fc in serum free DMEM for 1 hour at 37°C. The medium containing pseudovirus with or without PD-CLD-Fc was added to wells with a multiplicity of infection (MOI) of 0.4 and incubated for 2 hours at 37°C 5% CO_2_ before being removed and replaced with fresh culture medium. Twenty-four hours later, cells were harvested for flow cytometry analysis, with floating cells harvested as per the Luciferase assay above. The remaining cell monolayer was detached using 50 μl of 1× Trypsin-EDTA (Thermo Fisher Scientific; 25300-054), incubated for 5 min at 37°C, and neutralized with culture media, and detached cells were transferred to the round-bottom 96-well plate, with an additional wash to harvest the remaining cells. Cells were pelleted by centrifugation at 400*g* for 10 min and resuspended in PBS containing 1% paraformaldehyde (PFA, Ted Pella) and incubated overnight at 4°C. Before analysis, cells were pelleted down by centrifugation, PBS-PFA 1% was removed, and pellets were resuspended in fluorescence-activated cell sorting buffer. Cells were analyzed using a five-laser Aurora with a plate loader (Cytek), followed by manual gating in FlowJo 10 (Becton-Dickinson). Percent infection is expressed as a function of percentage of Zs-Green–positive cells detected in infected wells not treated with PD-CLD-Fc set to 100% and uninfected cells as baseline.

### Assays with authentic SARS-CoV-2 and A549-ACE2 cells

A549 cells overexpressing ACE2 were seeded in poly-l-lysine–coated 96-well plates at a density of 2.5 × 10^4^ cells per well and incubated overnight at 37°C. The next day, SARS-CoV-2 (WA01 isolate) and SARS-CoV-2 Omicron variant (DPH P2 isolate) were preincubated separately with different concentrations of PD-CLD-Fc or monoclonal antibodies for 1 hour at 37°C and then added to the cells (final MOI = 0.5). Twenty-four hours later, the cells were harvested to assess N-protein expression by immunofluorescence. Medium was removed, and cells were fixed with 10% formalin for 30 min. Fixed cells were permeabilized for 15 min with 0.1% Triton-X and then blocked for 1 hour with 10% goat serum and 1% BSA at room temperature (RT). Cells were incubated with SARS-CoV nucleoprotein (N) antibody (Rockland; 200-401-A50, 1:2000) at 4°C overnight. Afterward, cells were incubated with goat anti-rabbit Alexa Fluor 568 secondary antibody (Invitrogen; A-11011, 1:1000) at RT for 1 hour in the dark. Cells were counterstained with 4′,6-diamidino-2-phenylindole (1:1000) and visualized using an EVOS M5000 imaging system (Thermo Fisher Scientific). Images were processed using ImageJ (National Institutes of Health) to determine the percentage of N protein–positive cells in a given treatment sample. This was then normalized by the percentage of N^+^ cells in samples that were not treated with drug to determine the percentage of infection relative to the untreated sample.

### Assays with authentic SARS-CoV-2 and iAT2 cells

Induced pluripotent stem cell–derived iAT2s of clone SPC2-ST-B2 were generated as previously described (*64*–*66*). To establish air-liquid interface cultures, iAT2s were seeded on 6.5-mm Transwell inserts (Corning; 3470) at 520,000 live cells/cm^2^. After 48 hours, apical medium was removed, and fresh basolateral medium was added every 48 to 72 hours. For SARS-CoV-2 infections, SARS-CoV-2 virus particles (Washington isolate SARS-CoV-2 USA_WA1/2020) were preincubated with the compounds (diluted in PBS) for 1 hour at 37°C. The virus-compound mixtures were then added onto the apical side of the Transwells at an MOI of 5. Following 2 hours of incubation with the cells at 37°C, the virus inoculum was removed, and the cells were maintained at 37°C for another 24 hours before they were harvested for flow cytometry analysis. For harvest, 200 μl of Accutase (Sigma-Aldrich; A6964) was added to the apical side of the cells and incubated at 37°C for 30 min. The dissociated cells were collected in Eppendorf tubes, pelleted by centrifugation at 300*g* for 5 min at 4°C, and resuspended in 4% PFA. The cells were fixed in PFA for 3 hours at 4°C, pelleted by centrifugation, resuspended in 1× PBS, and removed from biosafety level 3. Fixed iAT2s were permeabilized with saponin buffer (BioLegend; 421002), incubated with SARS-CoV N antibody (1:1000; Rockland, 200-401-A50) on ice for 30 min, and subsequently incubated with a donkey anti-rabbit secondary antibody (1:500; Jackson ImmunoResearch, 711-545-152) on ice for 30 min before flow cytometry analysis.

### SARS-CoV-2 studies in hamsters

In vivo studies were performed in Animal Biosafety Level 3 facilities at Colorado State University. All work was conducted under protocols approved by the Institutional Animal Care and Use Committee at Colorado State according to guidelines set by the Association for the Assessment and Accreditation of Laboratory Animal Care and the U.S. Department of Agriculture. Eight-week-old Syrian hamsters obtained from Charles River Laboratories or Envigo were housed four per cage under Animal Biosafety Level 3 containment. In the prophylaxis model, groups of hamsters were treated with PD-CLD-Fc, PD-Fc, PD-Li-Fc, Fc-Li-PD, or PBS via intraperitoneal injection beginning 8 hours before viral challenge. A dose of 40 mg/kg per day was administered with a treatment of PD-CLD-Fc every 24 hours (−8, 16, and 40 hours) or 20 mg/kg treatments of PD-Fc, PD-Li-Fc, or Fc-Li-PD every 12 hours (−8, 4, 16, 28, and 40 hours). In the therapy model, a single treatment of PD-CLD-Fc or PBS (150 mg/kg) was administered via intraperitoneal injection at 12 hours after viral challenge. Virus challenge was conducted under ketamine-xylazine anesthesia by intranasal instillation of 100 μl of SARS-CoV-2 (strain SARS-CoV-2/human/USA/WA-CDC-WA1/2020 isolate) obtained from BEI Resources and passaged once in Vero and once in Vero E6 cells; the dose determined by back-titration of the inoculum was 1.1 × 10^4^ PFU. Hamsters were evaluated clinically and weighed once daily. An equal number of hamsters from each treatment group was euthanized and necropsied 3 or 4 days after challenge, depending on the study. For animals euthanized on these days, a sample of ∼100 mg of right cranial and right caudal lung lobes was excised, immersed in 0.9 ml of BA1/FBS, and homogenized using a mixer mill with stainless steel balls. Tissue homogenates were frozen to −80°C until assay. Virus titrations were performed using a double-overlay plaque assay on Vero E6 cells in six-well plates. Briefly, serial 10-fold dilutions of tissue homogenate samples were inoculated onto drained monolayers and incubated for 45 min, and 2 ml of a first overlay (0.5% agarose in minimum essential medium) without neutral red was added to each well. One day later, a second 2-ml overlay containing neutral red (0.06 mg/ml) was added to each well, and plaques were counted 1 and 2 days later. For histopathologic analysis, nasal turbinates and lungs were dissected and placed in 10% buffered formalin before histological sectioning. Histopathology was scored blinded by a board-certified veterinary pathologist.

### Enzyme-linked immunosorbent assays

To measure binding to S1 proteins from SARS-CoV-2 variants, 96-well ELISA plates were coated with PD-CLD-Fc protein at 5 μg/ml in PBS and incubated overnight at 4°C. The plates were washed and blocked (PBS, 5% milk) for 1 hour at RT. SARS-CoV-2 Spike S1-His recombinant protein (WT D614G; 40591-V08H3, Alpha B.1.1.7; 40591-V08H12, Beta B.1.351; 40591-V08H10, Delta B.1.617.2; 40591-V08H23, Epsilon B.1.427; 40591-V08H17, Gamma P.1; 40591-V08H14, Omicron B.1.1.529; and 40591-V08H41, all from Sino Biological) was added, diluted in PBS + 1% BSA (assay buffer) at concentrations varying from 0.0001 to 25 μg/ml, and incubated for 30 min at RT. The mutations included in each recombinant S1 protein are detailed in table S2. After washing, bound SARS-CoV-2 Spike S1-His recombinant protein was detected with mouse anti–His-tag-HRP (SouthernBiotech; 4603-05) diluted 1:5000 in assay buffer.

To measure binding to Fcγ receptors, 96-well ELISA plates were coated with recombinant human FcγRI (R&D Systems; 1257-FC), FcγRIIa (R&D Systems; 9595-CD), or FcγRIIIa protein (R&D Systems; 4325-FC) at 0.2 μg/ml in PBS and incubated overnight at 4°C. The following day, plates were washed and blocked (PBS, 5% milk) for 1 hour at RT. Subsequently, plates were incubated for 30 min with ACE2-Fc proteins or polyclonal human IgG (BioXCell; BE0092) diluted in assay buffer (PBS, 1% BSA) at concentrations varying from 0.1 to 500 μg/ml. After washing, bound ACE2-Fc protein was detected with F(ab’)_2_ goat antihuman IgG-HRP (Southern Biotech; 2042-05), diluted 1:5000 in assay buffer.

To measure binding to C1q, 96-well ELISA plates were coated with ACE2-Fc proteins or polyclonal human IgG (BioXCell; BE0092) diluted in PBS at concentrations varying from 10 to 0.005 μg/ml and incubated overnight at 4°C. The day after, plates were washed and blocked with assay buffer for 1 hour at RT. Recombinant human C1q protein (Millipore Sigma; C1740-1MG) was added to the plates at 5 μg/ml in assay buffer and incubated for 30 min at RT. The plates were washed, and polyclonal rabbit antihuman C1q (Agilent Dako; A0136) was added, diluted 1:1000 in assay buffer, and incubated for 15 min at RT and then washed. C1q bound to ACE2-Fc or human IgG was detected with goat anti-rabbit Ig (human adsorbed)–HRP (SouthernBiotech; 4010-05) diluted 1:5000 in assay buffer.

To compare the ability of PD-CLD-Fc to inhibit the binding of WT and PFS S-protein trimer to immobilized ACE2, a competitive ELISA was performed. Recombinant WT (Sino Biological; 40589-V08B1) or PFS S-protein trimer (LakePharma) was incubated at 0.1, 1, or 10 μg/ml with 5 μg/ml trypsin-EDTA (Thermo Fisher Scientific; 25300-054) and varying concentrations of PD-CLD-Fc (0 to 50 μg/ml) in PBS + 1% BSA assay buffer for 15 min at 37°C. Subsequently, the samples were transferred to ELISA plates that had been precoated with ACE2 (2 μg/ml) (the PD-Fc ACE2-Fc was used) in PBS overnight and blocked (PBS, 5% milk) for 1 hour at RT. After 30 min of sample incubation at RT, the plates were washed, and the bound S-protein trimer was detected with mouse anti–His-tag-HRP (SouthernBiotech; 4603-05) diluted 1:5000 in assay buffer.

To detect the bound S-protein trimer/PD-CLD-Fc complex, the recombinant purified WT S-protein trimer (Sino Biological; 40589-V08B1) or the PFS S-protein trimer (LakePharma) was incubated at 1 μg/ml with trypsin-EDTA (5 μg/ml; Thermo Fisher Scientific; 25300-054) and varying concentrations of PD-CLD-Fc (0 to 50 μg/ml) in assay buffer for 15 min at 37°C. Subsequently, the samples were transferred to ELISA plates that had been precoated with anti–SARS-CoV-2 S protein S2 antibody (2 μg/ml; BioLegend, 943202) in PBS overnight and blocked (PBS, 5% milk) for 1 hour at RT. After 30 min of sample incubation at RT, the plates were washed, and the bound S-protein trimer-PD-CLD-Fc complex was detected with HRP-conjugated goat anti-human IgG Fc, multispecies adsorbed (SouthernBiotech; 2014-05), and diluted 1:5000 in assay buffer.

For all ELISAs, 3,3′,5,5′-Tetramethylbenzidine substrate was used for color development, and the reactions were stopped with 1 M phosphoric acid. The optical density was measured at 450 nm on a SpectraMAX 190 Microplate reader.

### S-protein pulldown and Western blotting

The recombinant purified WT S-protein trimer (1 μg/ml; Sino Biological; 40589-V08B1) or the PFS S-protein trimer (LakePharma) was incubated with trypsin-EDTA (5 μg/ml; Thermo Fisher Scientific; 25300-054) and varying concentrations of PD-CLD-Fc (0 to 10 μg/ml) in assay buffer (PBS, 1% BSA) for 15 min at 37°C. Subsequently, the samples were incubated with His-Tag Isolation Dynabeads (Invitrogen, Thermo Fisher Scientific; 10103D) according to the manufacturer’s instructions to separate the His-tagged protein–containing fraction and the non–His-tagged protein–containing fraction. The samples were placed on a DynaMag magnet (Invitrogen, Thermo Fisher Scientific; 10103D), and the supernatants containing the non–His-tagged protein fraction were collected. The His-tagged protein-bead complexes were washed three times in PBS and 0.01% Tween 20, followed by imidazole elution in PBS and 0.01% Tween 20 containing 0.3 M imidazole (Sigma-Aldrich; 68268). The samples were then placed on the magnet, and the supernatants containing the eluted His-tagged proteins were collected. After mixing with Laemmli sample buffer (Bio-Rad; 161-0747), the samples were boiled for 5 min at 95°C and loaded and run on a 10% Mini-PROTEAN TGX precast protein gel (Bio-Rad; 4561094). The proteins were transferred onto a nitrocellulose membrane, which was subsequently blocked [tris-buffered saline and 0.01% Tween 20 (TBST) buffer and 5% milk] for 1 hour at RT and stained overnight at 4°C with primary antibody, anti–SARS-CoV-2 S protein S1 antibody (BioLegend; 944401), or anti–SARS-CoV-2 S protein S2 antibody (BioLegend; 943202), diluted 1:1000 and 1:5000 in TBST and 1% BSA, respectively. The following day, the blots were washed 3 × 5 min in TBST buffer and stained for 1 hour at RT with either mouse anti-rat kappa-HRP (SouthernBiotech; 3090-05) or rat anti-mouse kappa-HRP (SouthernBiotech; 1170-05) both diluted 1:5000 in TBST and 1% BSA for detection of S protein S1 and S2, respectively. The blots were washed for 3 × 5 min in TBST buffer and then developed with SuperSignal West Pico PLUS Chemiluminescent Substrate (Thermo Fisher Scientific; 34579). Blots were imaged on a ChemiDoc MP Imaging System (Bio-Rad). Measurement of band intensity (densitometry) was performed using GNU Octave version 6.4. In brief, lanes from each Western blot image were manually selected, and an electropherogram was created for each lane by calculating the mean image signal for each horizontal line within the vertical lane above the average photographic background signal of the image. Peaks corresponding to the bands of interest were then manually selected within the electropherogram, and the area under the curve for each peak was calculated and used as the band intensity.

### ACE2 enzymatic activity assay

To assess the ACE2 enzymatic activity of ACE2-Fc proteins, a fluorogenic substrate was used. ACE2 cleaves the Mca-YVADAPK(Dnp)-OH (R&D Systems; ES007) peptide at the C-terminal lysine residue. Cleavage liberates the Dnp (2,4-dinitrophenyl) moiety that quenches the fluorescence of the Mca (7-methoxycoumarin) moiety, resulting in increased fluorescence (*50*, *67*). The experiment was performed in triplicate. For each replicate, serial threefold dilutions of each ACE2-Fc were made in reaction buffer (0.1% BSA, 1 M sodium chloride, and 0.5 mM zinc chloride at pH 7.5) in a Black 96-well MaxiSorp Immuno Plate (Thermo Fisher Scientific; 437111). Mca-YVADAPK(Dnp)-OH substrate was then added to each well to achieve a concentration of 20 μM. The plate was placed immediately in a SpectraMax M3 microplate reader (Molecular Devices) that had been prewarmed to 37°C, and fluorescence was monitored at an excitation wavelength of 328 nm and an emission wavelength of 392 nm at 10-s intervals for 5 min. Maximum velocity for the substrate cleavage reaction in each well was calculated by SoftMax Pro software (Molecular Devices).

### Statistical analysis

Statistical analysis was performed using Prism 9 (GraphPad Software). Error bars denote the SEM. Comparison of body weight change over time between groups was performed via a mixed-effects model using the maximum likelihood method with Geisser-Greenhouse correction and correcting for multiple comparisons using the Šidák method, as advised by Prism. Comparisons of viral titers and lung histopathology scores were performed by two-sided *t* test. A *P* value of less than 0.05 was considered to be statistically significant.

## Notes

View/request a protocol for this paper from *Bio-protocol*.
