# Supplementary material for: Optimized ACE2 decoys neutralize antibody-resistant SARS-CoV-2 variants through functional receptor mimicry and treat infection in vivo
Source: Sci Adv. 2022 Dec 7;8(49):eabq6527. doi: 10.1126/sciadv.abq6527 (PMC9728973; doi:10.1126/sciadv.abq6527)
Supplement: Supplementary file 1 — Figs. S1 to S7 Tables S1 and S2 [file sciadv.abq6527_sm.pdf]

Science Advances
AAAS

Supplementary Materials for

Optimized ACE2 decoys neutralize antibody-resistant SARS-CoV-2 variants through functional receptor mimicry and treat infection in vivo

James A. Torchia *et al.*

Corresponding author: Gordon J. Freeman, gordon_freeman@dfci.harvard.edu

Sci. Adv. , eabq6527 (2022)
DOI: 10.1126/sciadv.abq6527

This PDF file includes:

Figs. S1 to S7
Tables S1 and S2

Supplementary Materials

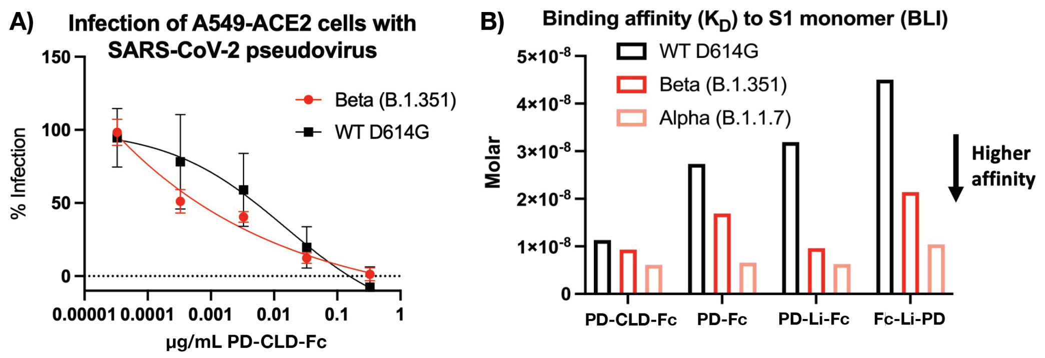

Fig. S1. ACE2-Fc decoy receptors bind and neutralize variants of concern. (**A**) Neutralization by PD-CLD-Fc of infection of ACE2-transduced A549 cells by VSV particles pseudotyped with D614G or Beta variant SARS-CoV-2 S-protein. Cells were infected with an MOI of 0.4. (**B**) The binding of soluble S1 monomer from the D614G parent, Alpha (B.1.1.7), and Beta (B.1.351) variants to immobilized ACE2-Fc was measured by biolayer interferometry (BLI).

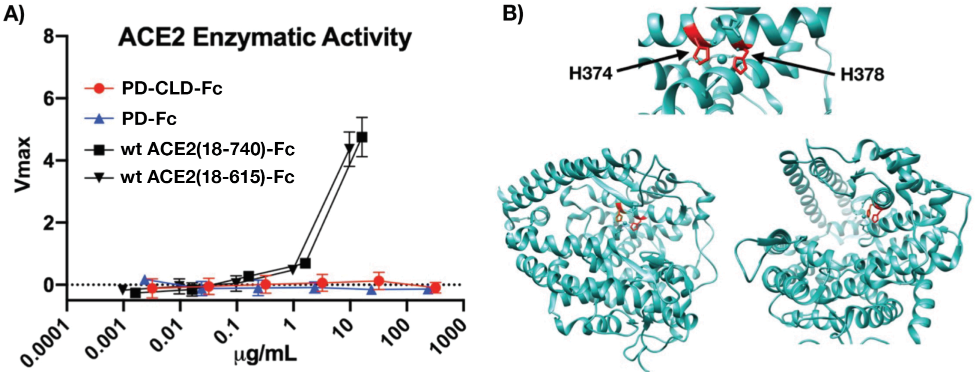

Fig. S2. ACE2 enzymatic activity. (**A**) Enzymatic activity of the PD-CLD-Fc and PD-Fc ACE2-Fc fusion proteins was assessed by monitoring cleavage of a fluorogenic peptide substrate. Enzymatic activity was compared to that of control ACE2-Fc fusions (wt) with no modifications in the ACE2 PD. (**B**) Renderings of the ACE2 PD highlighting residues H374 and H375, which are responsible for chelating a zinc atom necessary for ACE2 enzymatic activity. These residues are located deep within the angiotensin II binding cleft.

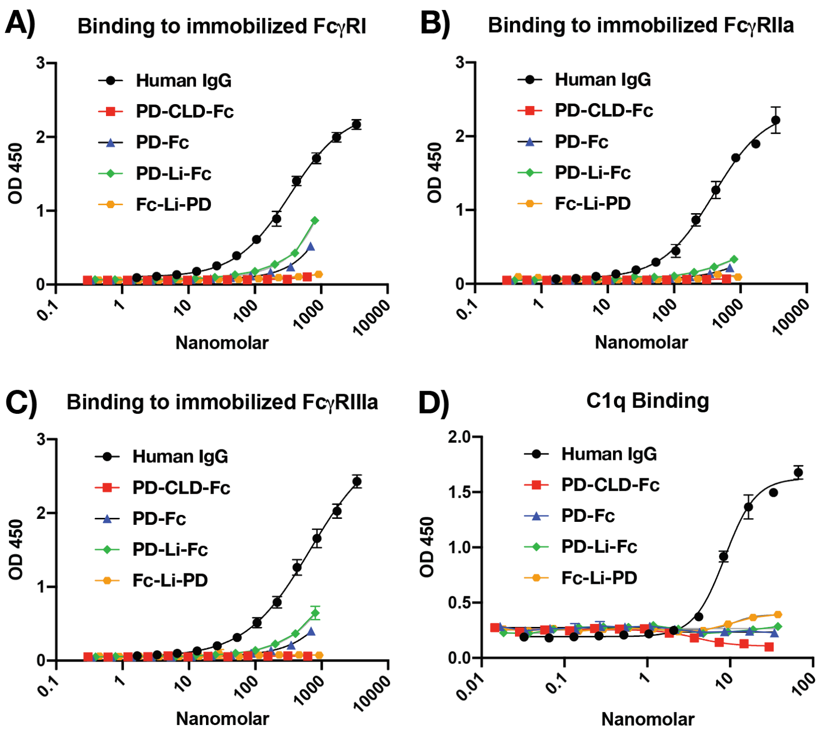

Fig. S3. FcγR and C1q binding. Binding of soluble ACE2-Fc compounds or polyclonal human IgG to immobilized FcγRI (**A**), FcγRIIa (**B**), or FcγRIIIa (**C**). (**D**) Binding of soluble C1q to immobilized ACE2-Fc compounds or polyclonal human IgG.

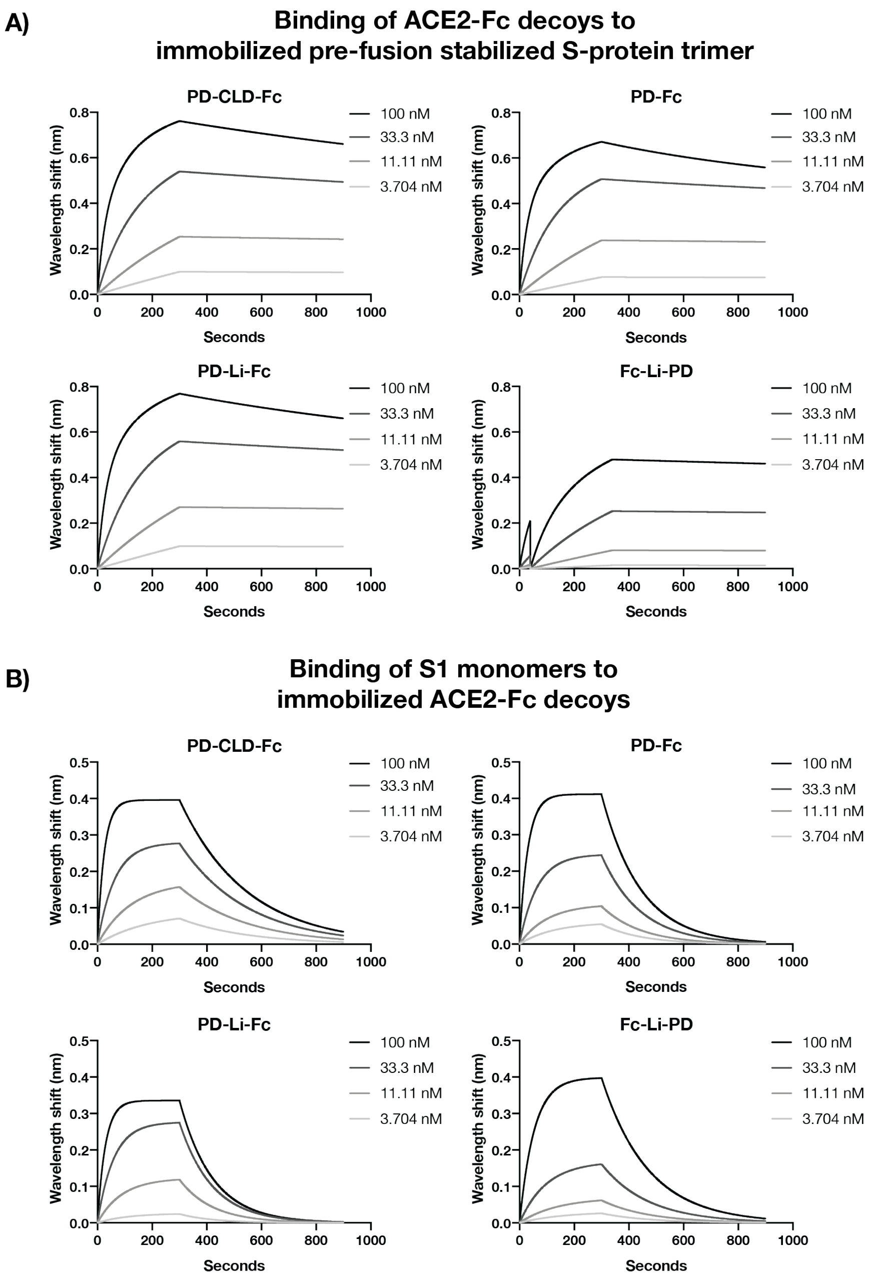

Fig. S4. Binding of ACE2-Fc decoys to PFS S-protein trimer and S1 monomers by BLI. (**A**) BLI tracings representing the binding of soluble ACE2-Fc decoys to immobilized pre-fusion stabilized S-protein trimer. (**B**) BLI tracings representing the binding of soluble S1 monomer to immobilized ACE2-Fc decoys was measured by BLI.

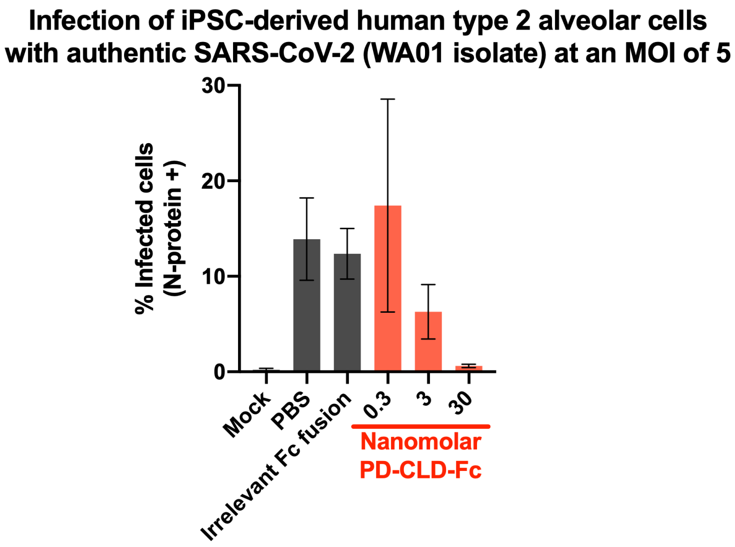

Fig. S5. ACE2 decoys inhibit authentic SARS-CoV-2 infection of iAT2 human respiratory epithelium

We evaluated neutralization of infection of human induced pluripotent stem cell (iPSC)-derived alveolar type II epithelial cells (iAT2) grown in an air-liquid interface by authentic SARS-CoV-2 virus (WA01 isolate). Viral nucleocapsid (N)-protein expression levels were assessed by flow cytometry of fixed and permeabilized iAT2 cells and used as a metric of SARS-CoV-2 infection.

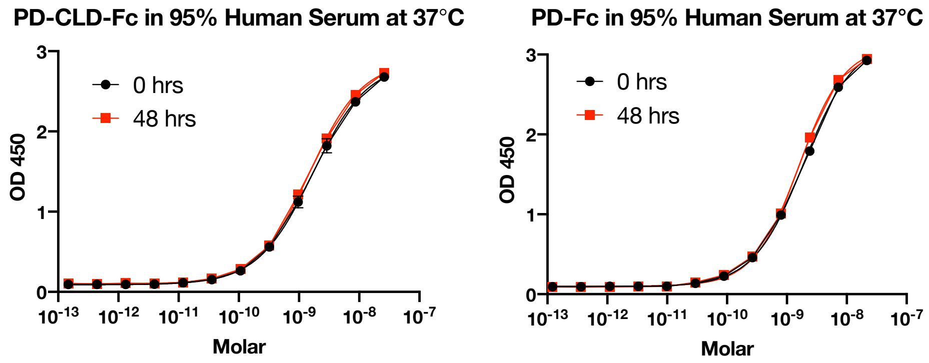

Fig. S6. Serum stability. PD-CLD-Fc and PD-Fc were incubated in 95% human serum for 0 hours (on ice) or at 37°C for 48 hours. After incubation, serial dilutions were performed in plates coated with pre-fusion stabilized S-protein trimer. After thorough washing of the plates, binding of the ACE2-Fc proteins was detected by an anti-human IgG-HRP antibody. Because the immobilized S-protein binds to the ACE2 PD and the detection antibody binds to the Fc domain on the opposite end of the ACE2-Fc molecule, the integrity of the ACE2-Fc molecule can be assessed by this sandwich ELISA.

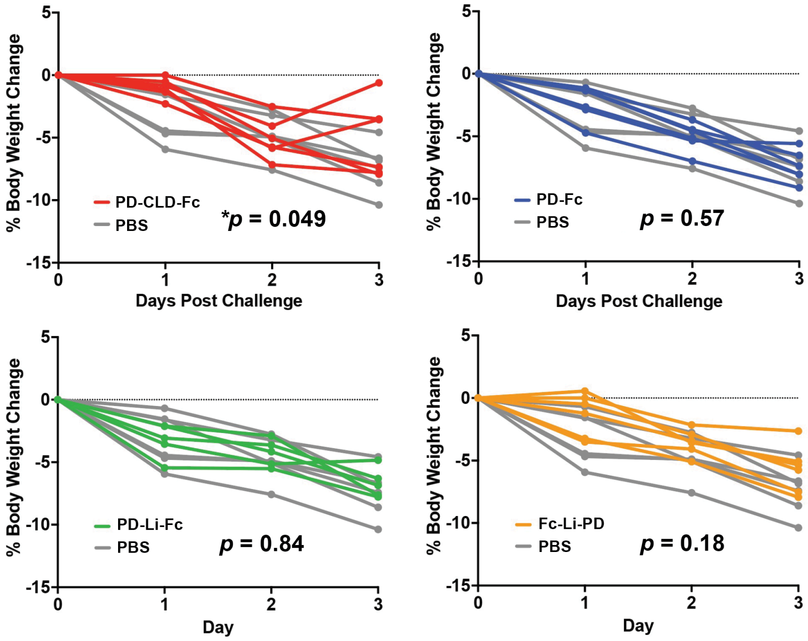

Fig. S7. Weight loss in the prophylaxis experiment. Syrian hamsters were challenged intranasally with $1x10^4$ PFU of the WA01 isolate of SARS-CoV-2 and weighed daily. Animals were treated with a total of 40 mg/kg/day or vehicle (PBS) for three days. Treatment began eight hours prior to viral challenge. All animals were sacrificed on day 3.

Endpoint	Statistical test	Experiment	Treatment	Mean	Treatment vs. Placebo Difference Mean (SEM)	*p* value
Body Weight Change (Percent)	MMRM*	Prophylactic	PD-CLD-Fc	-2.80	1.06 (0.70)	0.049
			PD-Fc	-3.71	0.15 (0.62)	0.57
			PD-Li-Fc	-3.55	0.31 (0.63)	0.85
			Fc-Li-PD	-2.64	1.21 (0.70)	0.18
			Placebo	-3.86		
		Therapeutic	PD-CLD-Fc	-4.00	3.00 (0.57)	<0.0001
			Placebo	-7.00		
Nasal turbinate viral titer log(PFU/100 mg)	Two-tailed T-test	Prophylactic	PD-CLD-Fc	5.58	-2.01 (0.50)	0.0024
			PD-Fc	7.48	-0.11 (0.38)	0.78
			PD-Li-Fc	6.76	-0.83 (0.44)	0.094
			Fc-Li-PD	7.90	0.30 (0.28)	0.31
			Placebo	7.59		
		Therapeutic	PD-CLD-Fc	6.01	-0.27 (0.26)	0.31
			Placebo	6.28		
Lung viral titer log(PFU/100 mg)	Two-tailed T-test	Prophylactic	PD-CLD-Fc	6.40	-0.86 (0.33)	0.015
			PD-Fc	7.24	-0.017 (0.25)	0.95
			PD-Li-Fc	6.93	-0.33 (0.38)	0.40
			Fc-Li-PD	7.18	-0.083 (0.27)	0.76
			Placebo	7.26		
		Therapeutic	PD-CLD-Fc	6.25	-0.15 (0.33)	0.67
			Placebo	6.40		
Lung pathology score	Two-tailed T-test	Therapeutic	PD-CLD-Fc	30.6	-4.5 (2.1)	0.043
			Placebo	35.1		

* Mixed Model for Repeated Measures (MMRM) using Geisser-Greenhouse correction and Sidak's multiple comparisons test as implemented in Prism 9

Table S1. Summary of statistical analysis of the hamster studies.

Variant	Mutations in the recombinant S1 protein
Alpha B.1.1.7	HV69-70 deletion, Y144 deletion, N501Y, A570D, D614G, P681H
Beta B.1.351	K417N, E484K, N501Y, D614G
Delta B.1.617.2	T19R, G142D, E156G, 157-158 deletion, L452R, T478K, D614G, P681R
Epsilon B.1.427	W152C, L452R, D614G
Gamma P.1	L18F, T20N, P26S, D138Y, R190S, K417T, E484K, N501Y, D614G, H655Y
Omicron B.1.1.529.1	A67V, HV69-70 deletion, T95I, G142D, VYY143-145 deletion, N211 deletion, L212I, ins214EPE, G339D, S371L, S373P, S375F, K417N, N440K, G446S, S477N, T478K, E484A, Q493R, G496S, Q498R, N501Y, Y505H, T547K, D614G, H655Y, N679K, P681H

Table S2. Mutations in recombinant variant S1 proteins
